# Refinement of protein structures using a combination of quantum-mechanical calculations with neutron and X-ray crystallographic data

**DOI:** 10.1107/S205979831900175X

**Published:** 2019-03-28

**Authors:** Octav Caldararu, Francesco Manzoni, Esko Oksanen, Derek T. Logan, Ulf Ryde

**Affiliations:** a Department of Theoretical Chemistry, Lund University, Chemical Centre, PO Box 124, SE-221 00 Lund, Sweden; b Department of Biochemistry and Structural Biology, Centre for Molecular Protein Science, Lund University, Chemical Centre, PO Box 124, SE-221 00 Lund, Sweden; c Instruments Division, European Spallation Source ESS ERIC, PO Box 176, SE-221 00 Lund, Sweden

**Keywords:** refinement, neutron crystallography, hydrogen atoms, quantum chemistry, galectin-3, lytic polysaccharide monooxygenase, quantum refinement

## Abstract

Neutron crystallography is a powerful method to determine the positions of H atoms in macromolecular structures. However, it is sometimes hard to judge what would constitute a chemically reasonable model, and the geometry of H atoms depends more on the surroundings (for example the formation of hydrogen bonds) than heavy atoms, so that the empirical geometry information for the H atoms used to supplement the experimental data is often less accurate. These problems may be reduced by using quantum-mechanical calculations. A method has therefore been developed to combine quantum-mechanical calculations with joint crystallographic refinement against X-ray and neutron data. A first validation of this method is provided by re-refining the structure of the galectin-3 carbohydrate-recognition domain in complex with lactose. The geometry is improved, in particular for water molecules, for which the method leads to better-resolved hydrogen-bonding interactions. The method has also been applied to the active copper site of lytic polysaccharide monooxygenase and shows that the protonation state of the amino-terminal histidine residue can be determined.

## Abstract 2

A method has been developed to combine quantum-mechanical calculations with joint crystallographic refinement against X-ray and neutron data. Applications to structures of the galectin-3C–lactose complex and lytic polysaccharide monooxygenase show that it can improve the geometry of hydrogen bonds, particularly those involving water molecules, as well as determine the protonation states of key residues.

## 1. Introduction   

The majority of structural information about biological macromolecules is obtained from X-ray crystallography. An important limitation of X-ray crystallography is that H atoms can only rarely be visualized in the electron-density maps, as the scattering by just one electron is too weak. Neutron crystallography (Blakeley, 2009 ▸) is a method that is able to complement the information from X-ray crystallography because neutrons are scattered by the nuclei. Moreover, deuterium, the heavier isotope of hydrogen, scatters neutrons comparably to carbon or nitrogen (Sears, 1986 ▸). Consequently, neutron structures can help to determine the positions of H atoms, describe the geometry of hydrogen bonds, determine the protonation and tautomeric states of ligands and pinpoint the function of water molecules. However, as the inclusion of H atoms in the model increases the number of parameters to be refined and neutron structures are typically at a rather low resolution, neutron data alone are typically not sufficient to define all of the atomic positions. Therefore, refinement protocols have been introduced that combine X-ray and neutron data into a single target function, against which the coordinates are refined (Coppens, 1967 ▸; Orpen *et al.*, 1978 ▸; Wlodawer, 1980 ▸; Wlodawer & Hendrickson, 1982 ▸; Adams *et al.*, 2009 ▸; Afonine *et al.*, 2010 ▸; Wlodawer & Sjölin, 1982 ▸; Fisher *et al.*, 2007 ▸).

At the resolutions typical for macromolecular structures, the quality and information content of the refined structural model critically depends on prior chemical knowledge about the system, such as the amino-acid sequence and a set of chemical restraints (Cruickshank, 1999 ▸). In practice, the latter information, in the form of a molecular-mechanics (MM) or statistical force field, is used to restrain the bond lengths, angles and dihedrals in refinement, as well as to ensure proper chirality and to avoid sterical clashes. For the non-H atoms in amino acids and other standard constituents of proteins, these values are well known from a large number of high-resolution crystal structures and are available in the refinement software (Engh & Huber, 1991 ▸). However, this is not the case for many non-protein groups, such as metal sites, substrates and inhibitors (Ryde *et al.*, 2002 ▸; Nilsson *et al.*, 2003 ▸; Zheng, Reimers *et al.*, 2017 ▸; Borbulevych *et al.*, 2016 ▸). Moreover, the geometrical information available for bonds involving H atoms is less reliable than for heavier atoms, even for protein components, not to mention non-protein compounds.

The restraints used in crystallographic refinement provide quite a crude description of the molecular system, as they are based on an average over many structures. The chemical environment may influence the bond lengths and angles, for example by electrostatic and induction effects, in ways that are not captured by typical restraints. The ideal geometry of hydrogen-containing bonds is particularly sensitive to the local chemical environment, which makes an averaging approach less attractive with hydrogen.

To address these issues in X-ray crystallography, we have previously developed a method to locally replace the MM restraints in the refinement by more accurate quantum-mechanical (QM) calculations: the quantum-refinement approach (Ryde *et al.*, 2002 ▸; Ryde & Nilsson, 2003 ▸; Ryde, 2007 ▸; Genoni *et al.*, 2018 ▸). It is in principle a standard crystallo­graphic refinement in which the MM restraints are replaced by more accurate QM calculations for a small part of the protein of particular interest, such as an active site. From a computational chemistry viewpoint, it can be seen as a combined QM/MM calculation restrained by the crystallographic raw data. We showed that crystal structures can be locally improved using such a method, the protonation states of metal-bound water molecules can be determined and the photoreduction of metal sites can be identified (Nilsson & Ryde, 2004 ▸; Söder­hjelm & Ryde, 2006 ▸; Rulíšek & Ryde, 2006 ▸; Ryde & Nilsson, 2003 ▸). The approach has been implemented in several software programs by other groups (Yu *et al.*, 2005 ▸; Hsiao *et al.*, 2010 ▸). In particular, Merz and Westerhoff have developed methods to combine X-ray crystallographic refinement with linear-scaling semi-empirical calculations of the whole protein and have shown that protonation states and ligand-binding conformations can be determined (Borbulevych *et al.*, 2014 ▸, 2016 ▸; Reynolds, 2014 ▸; Yu *et al.*, 2005 ▸). The *Q*|*R* project also aims at refining macromolecular structures using QM methods (Zheng, Moriarty *et al.*, 2017 ▸; Zheng, Reimers *et al.*, 2017 ▸).

In this paper, we extend the quantum-refinement approach to neutron data, as well as combined X-ray and neutron refinement. The method is implemented in the freely available *nCNS* software (Adams *et al.*, 2009 ▸). As test cases, we apply the method to the refinement of the ligand-binding site of the carbohydrate-recognition domain of galectin-3 with a bound lactose molecule (Manzoni *et al.*, 2018 ▸) and to the active copper site of lytic polysaccharide monooxygenase (LPMO).

Galectin-3 is a mammalian β-galactoside-binding protein involved in glycoprotein trafficking, signalling, cell adhesion, angiogenesis, macrophage activation and apoptosis (Leffler *et al.*, 2002 ▸; MacKinnon *et al.*, 2008 ▸; Delacour *et al.*, 2009 ▸; Liu & Rabinovich, 2010 ▸; Grigorian & Demetriou, 2010 ▸; Johannes *et al.*, 2018 ▸). It has been implicated in inflammation, immunity, cancer development and metastasis (Rabinovich *et al.*, 2007 ▸).

LPMO is a metalloenzyme that is capable of inserting a single O atom into the glycosidic C—H bond in polysaccharides (Vaaje-Kolstad *et al.*, 2010 ▸). This boosts polysaccharide decomposition, which could lead to a more energy-efficient production of biofuel from abundant polysaccharides such as cellulose (Harris *et al.*, 2014 ▸). LPMOs contain a copper ion coordinated by two histidine residues. One of the histidine residues coordinates bidentately to both the N-terminus and the imidazole side chain (Quinlan *et al.*, 2011 ▸).

We show that the combined QM/neutron/X-ray refinement works properly and gives rise to improved structures and to an easier interpretation of ambiguous data.

## 2. Methods   

### 2.1. Galectin-3   

Neutron data were collected to a resolution of 1.7 Å from a 1.8 mm^3^ crystal of perdeuterated carbohydrate-recognition domain of galectin-3 (galectin-3C) complexed with nondeuterated lactose using the LADI-III instrument at Institut Laue–Langevin, Grenoble, France (Blakeley *et al.*, 2010 ▸). X-ray data were collected to 1.0 Å resolution from the same crystal on beamline I911-3 at the MAX-II synchrotron, Lund, Sweden. Full details of data collection and processing have been presented in a previous article (Manzoni *et al.*, 2016 ▸). In that article, the neutron data set used here was referred to as ‘lactose-2’ and was 87.1% complete to 1.7 Å resolution (66.5% from 1.8 to 1.7 Å). The X-ray data are 99% complete to 1.0 Å resolution. However, in all refinements in this paper the X-ray data were truncated at 1.7 Å resolution. This was mainly performed because the program used for refinement, *nCNS* (see below), cannot refine anisotropic *B* factors. However, it also reflects a more common scenario than that which prevails for galectin-3C, namely that the X-ray and neutron diffraction limits are more similar. Furthermore, high-resolution X-ray data contain information on hydrogen positions that is potentially contradictory to the neutron data, as the peaks in the electron density are displaced towards the centre of the bond to the heavy atom, resulting in shorter bond lengths. A scenario with much higher resolution X-ray data was tested for LPMO. The finally published galectin-3C–lactose structure (Manzoni *et al.*, 2018 ▸), deposited in the PDB as entry 6eym, was refined against a more complete neutron data set (96% complete to 1.7 Å resolution) with higher multiplicity that was obtained by merging the data from two crystals (Manzoni *et al.*, 2016 ▸).

The starting structure for refinement was the 0.86 Å resolution X-ray structure of galectin-3 in complex with lactose with the water molecules removed (Saraboji *et al.*, 2012 ▸). The structure was first refined against the X-ray data using *REFMAC*5 (Murshudov *et al.*, 2011 ▸). Water molecules were added manually when visible at 1.7 Å resolution. Geometric restraints for the lactose molecule were generated in *phenix.elbow*. Joint X-ray/neutron refinement was then performed using *phenix.refine* (Afonine *et al.*, 2012 ▸). All calculations were performed on one core of an Intel Xeon E5-2650 processor at the LUNARC Aurora cluster at Lund University.

Upon the convergence of conventional joint refinement, the final structure was refined for 50 steps with the neutron crystallography patch of the *Crystallography and NMR System* (Brünger *et al.*, 1998 ▸) software (*nCNS*; Adams *et al.*, 2009 ▸) before QM refinement. We used the standard *nCNS* MM force field for all atoms (protein-allhdg_d.param and dod.param). The atom types and parameters used for lactose are presented in Supplementary Tables S2 and S3 (however, the lactose parameters will not affect the QM-refined results because they cancel exactly, as will be discussed below). After QM refinement, bulk-solvent correction and *B*-factor refinement were performed in *PHENIX*. Real-space difference density *Z*-scores (RSZDs) were calculated for selected structures with the *EDSTATS* module (Tickle, 2012 ▸) of the *CCP*4 software suite (Winn *et al.*, 2011 ▸).

**Figure 1:**
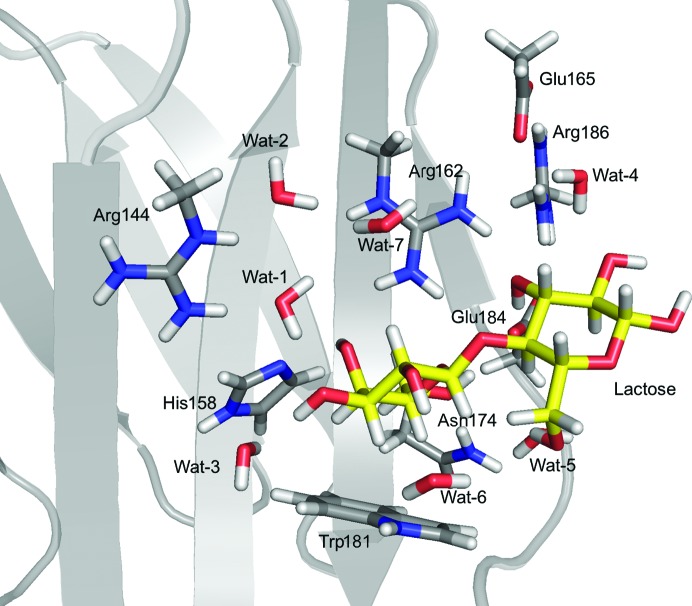
The QM system (S1 in Fig. 3 ▸) used in the *ComQum-U* calculations for galectin-3. All atoms involved in the QM calculation are shown as sticks, while the rest of the protein is shown as a cartoon and the remaining water molecules are hidden. Lactose is shown with yellow C atoms, whereas the protein residues are shown with grey C atoms.

The QM system for galectin-3 (Fig. 1 ▸) consisted of the lactose ligand as well as the side chains of Arg144, His158, Arg162, Glu165, Asn174, Trp181, Glu184 and Arg186, and seven water molecules (named Wat-1, Wat-2, Wat-3, Wat-4, Wat-5, Wat-6 and Wat-7 in the text; see Table 1 ▸ for the corresponding numbers in the deposited structure); 153 atoms in total. The QM system was selected to include all protein residues and water molecules that form hydrogen bonds to the lactose ligand, as well as one residue (Glu-165) and a few water molecules that connect these residues by hydrogen bonds. The Arg, His, Glu, Asn and Trp side chains were truncated in the QM system by replacing the CG, CB, CB, CA and CB atoms, respectively, by an H atom (the hydrogen link-atom approach), as implemented in the *ComQum-U* software (Ryde, 1996 ▸; Ryde & Olsson, 2001 ▸).

### 2.2. LPMO   

X-ray structure factors, coordinates, occupancies and *B* factors were obtained from the 1.1 Å resolution structure of AA10 LPMO (Bacik *et al.*, 2017 ▸; PDB entry 5vg0). Neutron structure factors were obtained from PDB entry 5vg1, a 2.1 Å resolution structure of the same AA10 LPMO (Bacik *et al.*, 2017 ▸). Prior to QM refinement, classical X-ray–neutron joint refinement was performed using *phenix.refine*, starting from the coordinates of the X-ray structure and adding D atoms with *phenix.ready_set*. D atoms at the N-terminal atoms were added manually. Full details of the classical joint refinement can be found in Caldararu *et al.* (2018 ▸). The same QM refinement procedure was then performed as described for galectin-3, except that the X-ray data were not truncated (*i.e.* all data to 1.1 Å resolution were kept in the re-refinements). However, anisotropic *B* factors were discarded as *nCNS* cannot handle these.

**Figure 2:**
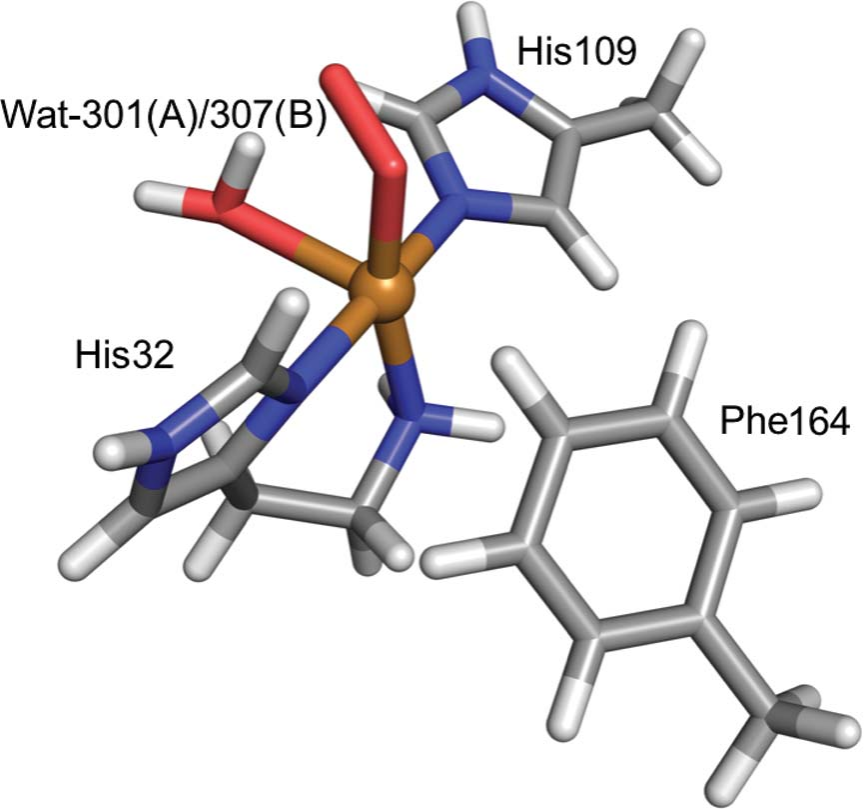
The QM system (S1 in Fig. 3 ▸) used in the *ComQum-U* calculations for LPMO.

The QM system for LPMO (Fig. 2 ▸) consisted of the first coordination sphere of the copper ion in either subunit *A* or *B* of the enzyme. It contained the copper ion, the imidazole ring of His109, the phenyl ring of Phe164, the full His32 residue, an O_2_-derived ligand bound to the Cu atom and a crystal water molecule (Wat-301 in subunit *A* and Wat-307 in subunit *B*). The three residues were truncated in the QM system by replacing the CB atoms or C atom (for His32) with H atoms, as described above. Based on a study of alternative oxidation states (Caldararu *et al.*, 2018 ▸), the copper ion was considered to be Cu^2+^, while the oxygen species was considered to be a peroxide, O^2^ ^2−^, resulting in a total net charge on the system of 0.

### 2.3. QM calculations   

The QM calculations were carried out using *TURBOMOLE* 7.0 (Furche *et al.*, 2014 ▸). The TPSS (Tao *et al.*, 2003 ▸) density functional theory (DFT) method and the def2-SV(P) basis set (Schäfer *et al.*, 1992 ▸) were used. Dispersion effects were included with the empirical DFT-D3 approach (Grimme *et al.*, 2010 ▸). This approach gives reasonable geometries for both biochemical and bioinorganic systems (Neese, 2006 ▸; Sure & Grimme, 2015 ▸; Antony *et al.*, 2015 ▸) and have been used in many applications in our group (Ryde, 2016 ▸).

## 3. Result and discussion   

### 3.1. Implementation   

The added value of QM calculations for crystallographic structure determination is to make the model consistent with both the crystallographic data and the QM energy function, avoiding the less accurate MM potential, and hence provide a chemically more reasonable model. QM is introduced for a small but interesting part of the structure in the QM/MM approach. Therefore, we introduce QM calculations only in the final steps of the structure-determination process, typically after a standard structure refinement. This will not affect the overall structure radically, but will provide better local geometries, help in resolving ambiguous interpretations of the density maps and support conclusions about unusual geometries.

We decided to implement the combined QM and neutron crystallographic refinement in the *nCNS* software (Adams *et al.*, 2009 ▸) for several reasons. Firstly, *nCNS* is freely available software, providing implementation of both neutron and joint neutron and X-ray refinement. Secondly, our previous quantum-refinement method was implemented in the *CNS* software (Brünger *et al.*, 1998 ▸), which made the extension to neutron crystallography straightforward. Thirdly, *CNS* was originally developed from the *CHARMM* MM software (Brooks *et al.*, 2009 ▸), meaning that it consists of an open symbolic language with existing implementation of MM force fields, as well as facile access to and manipulation of energies and forces, again strongly simplifying the implementation.

Crystallographic refinement is in principle a global pseudo-energy minimization using an energy function of the form
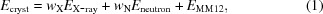
where *E*_MM12_ is an MM (or another empirical or statistical) energy function of the entire model (the meaning of the ‘12’ subscript will be apparent below when the total system is divided into two parts), whereas *E*_X-ray_ and *E*_neutron_ describe how closely the current model reproduces the experimental X-ray and neutron data, respectively. The latter could, in principle, be the crystallographic *R* factors, but typically more sophisticated maximum-likelihood refinement target functions are employed (Pannu & Read, 1996 ▸; Adams *et al.*, 1997 ▸). The two weight factors *w*_X_ and *w*_N_ are needed because *E*_MM12_ is typically in energy units, whereas the other two terms are plain numbers. They determine the relative weights of the three (pseudo-)energy terms so that, for example, setting *w*_X_ = 0 implies that a pure neutron crystallographic refinement is performed. Ideal values of the weight factors can be obtained by optimization of the *R*_free_ factor, but this is tedious for the joint energy function and therefore they are typically determined so that the three energy terms have a similar magnitude in a short MD simulation (Brünger & Rice, 1997 ▸; Brünger *et al.*, 1989 ▸; Adams *et al.*, 1997 ▸).

QM calculations can be introduced into this energy function by simply replacing *E*_MM12_ with a standard QM/MM energy function (Ryde, 2016 ▸). In this, QM is employed for a restricted, but interesting, part of the protein, called system 1 or the QM system. MM is employed for the remaining atoms in the considered model, called system 2 (and therefore the entire system will be called 12, as in *E*_MM12_). Within the subtractive QM/MM approach (Cao & Ryde, 2018 ▸), this is obtained in the following way:
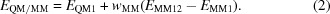



Here, *E*_QM1_ is the QM energy of system 1 and *E*_MM1_ is the MM energy of the same system. The latter ensures that no energy terms are double-counted by cancelling the MM terms of the QM system in *E*_MM12_ (therefore, the results will not depend on the MM parameters employed for the QM system). *w*_MM_ is a scale factor, which is needed because the statistics-based force field in *CNS* (Engh & Huber, 1991 ▸) typically gives energies that are approximately three times larger than an energy-based force field (Ryde *et al.*, 2002 ▸). Therefore, *w*_MM_ was always set to 1/3. In standard QM/MM with energy-based force fields, *w*_MM_ = 1 and is therefore normally omitted (Ryde, 2016 ▸).

Replacing *E*_MM12_ in (1) with *E*_QM/MM_ in (2) gives the final combined energy function:
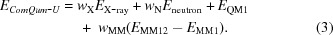



Forces were obtained from this energy function by using analytical differentiation and employing the chain rule for the hydrogen link-atoms (Ryde, 1996 ▸; Ryde & Olsson, 2001 ▸).

**Figure 3:**
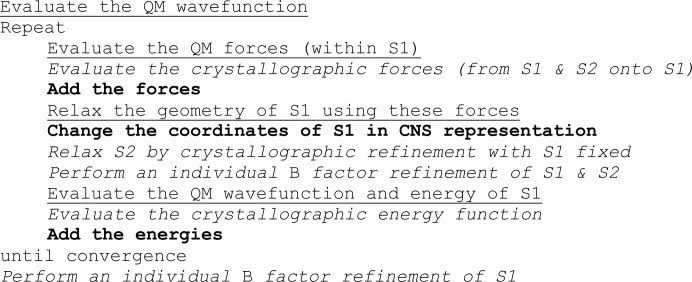
Flow chart of the *ComQum-U* program. S1 and S2 denotes systems 1 and 2. Steps in bold constitute the actual *ComQum-U* interface. Steps in italics are performed by the crystallographic refinement program (*nCNS*), whereas those that are underlined are run by the QM program. The whole procedure is driven by a Linux shell script.

The flow chart of the *ComQum-U* program is shown in Fig. 3 ▸. It is implemented using the *nCNS* xn_minimize.inp and xn_bindividual.inp files, with some simple modifications to write out crystallographic energies and forces. This ensures that all normal crystallographic manipulations and calculations are performed, for example bulk-solvent corrections and calculations of *R* factors. Moreover, it was necessary to read in and write out coordinates with a higher precision than standard PDB files to avoid convergence problems (Ryde *et al.*, 2002 ▸). For calculation of the crystallographic energies and forces the number of minimization steps was set to zero, whereas in the relaxation step it was set to one. The maximum-likelihood refinement target using amplitudes was employed. A simple *CNS* script was also employed to calculate the *E*_MM1_ energy term. The whole quantum-refinement procedure is driven by a Linux shell script, which is based on the *TURBOMOLE* geometry-optimization script *jobex* (Furche *et al.*, 2014 ▸). Relaxation of the QM system is performed by the *relax* program in *TURBOMOLE*, employing a Broyden–Fletcher–Goldfarb–Shanno quasi-Newton approach. Geometry optimizations were continued until the energy change between two iterations was less than 2.6 J mol^−1^ (10^–6^ arbitrary units) and the maximum norm of the Cartesian gradients was below 10^–3^ arbitrary units. A further description of the procedure can be found at http://signe.teokem.lu.se/~ulf/Methods/comqum_u.html, and the interface can be provided by the authors upon request (but note that *nCNS* and *TURBOMOLE* need to be installed separately and that a licence for *TURBOMOLE* is required). A typical re-refinement of the galectin-3 test system required around 150 QM energy and gradient calculations, which took about 5 h on a single processor, whereas re-refinement of the smaller LPMO test system required only around 90 QM energy and gradient calculations, which took about 2 h on a single processor.

Standard crystallographic refinement is performed without any electrostatic MM term. For X-ray data this is natural because the positions of the H atoms are normally unknown and the electrostatics of hydrogen bonds strongly depend on the hydrogen positions. For neutron structures, the positions of most (but typically not all) H (D) atoms are known, but the refinement is still traditionally performed without any electrostatics. To make the results compatible with the data obtained with standard refinement, we decided to exclude electrostatics from the calculations. However, it should be noted that electrostatics can easily be included in the energy function using MM atomic charges in *E*_MM12_ and a point-charge model of the protein in *E*_QM1_. The effect of electrostatics will be evaluated in a future study. Finally, it should be noted that QM calculations naturally involve electrostatics and the positions of all H atoms within the QM system need to be included.

### 3.2. Performance on galectin-3   

The performance of the *ComQum-U* program was examined by re-refining a preliminary, unpublished 1.7 Å resolution neutron structure of lactose bound to the perdeuterated carbohydrate-recognition domain of galectin-3. The structure was originally refined jointly against X-ray and neutron data from the same crystal to 1.7 Å resolution using *phenix.refine* (Afonine *et al.*, 2012 ▸) and was briefly re-refined with *nCNS* (Adams *et al.*, 2009 ▸) before running the quantum refinement. We included the lactose ligand, eight nearby amino-acid side chains and seven water molecules in the QM system, as shown in Fig. 1 ▸ (a total of 153 atoms).

We first checked that the program worked properly by refining the structure with varying values of the weight factors *w*_X_ and *w*_N_ in (1)–(3) [Disp-formula fd2] [Disp-formula fd3] from 0 to 10. By setting *w*_X_ = *w*_N_ = 0, we essentially obtain a QM/MM structure of the active site, although with the *CNS* MM force field and without any electrostatics. This gives a rather poor structure in terms of the crystallographic *R* factors (0.25/0.23, 0.21/0.19; these four values are *R*_free_/*R* first for the neutron data and then for the X-ray data).

**Figure 4:**
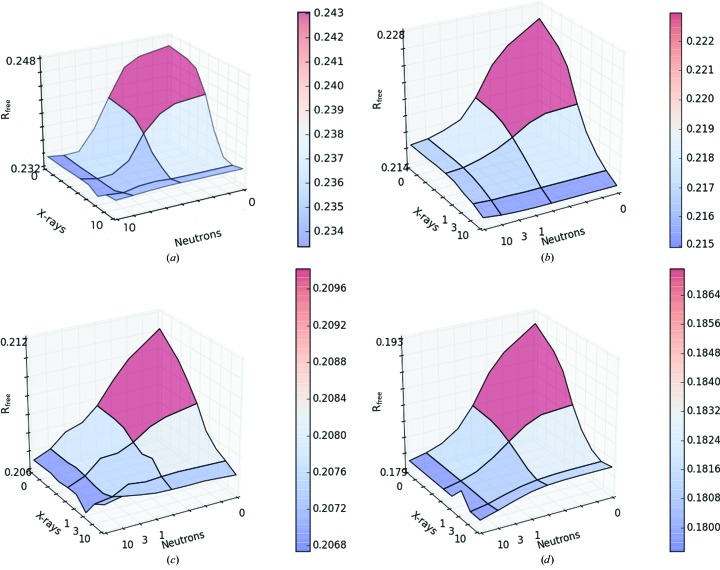
The dependence of the *R* factors on the weight factors *w*_N_ and *w*_X_ in the refinement of galectin-3 (see equation 3): (*a*) *R* ^N^_free_, (*b*) *R* ^N^, (*c*) *R* ^X^_free_ and (*d*) *R* ^X^. The raw data are given in Supplementary Table S1.

Next, we performed refinements with 8 × 8 different values of the weight factors *w*_X_ and *w*_N_ (0, 0.01, 0.03, 0.1, 0.3, 1, 3 and 10). The resulting *R* values are shown in Fig. 4 ▸ and Supplementary Table S1. It can be seen that the best *R* value for the neutron data (0.214) was obtained for the largest *w*_N_ = 10, as expected. It increased slightly when *w*_X_ was increased. Likewise, the best *R* value for the X-ray data (0.179) was obtained for the largest *w*_X_ = 10. However, the best values of *R*_free_ for both the neutron and X-ray data (0.233 and 0.207, respectively) are obtained at intermediate values at around *w*_N_ = 1 and *w*_X_ = 3. This shows that the refinement function works properly and that optimum values of the two weight factors can be obtained by optimizing the two *R*_free_ values, although the surfaces are quite flat. Performing an additional bulk-solvent correction and *B*-factor refinement in *PHENIX* decreased the *R*_free_ values to 0.179 for the X-ray data and 0.215 for the neutron data (Table 2 ▸), which is comparable to the value of 0.211 obtained from *phenix.refine* alone.

**Figure 5:**
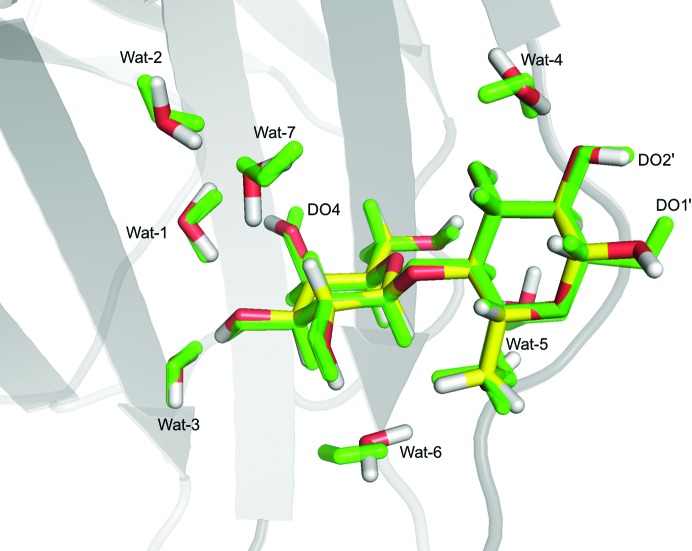
Structure before (green) and after (C atoms in yellow) QM refinement of the preliminary galectin-3 structure with *w*_N_ = 1 and *w*_X_ = 3. Protein residues are hidden for clarity (they basically do not move).

In Fig. 5 ▸, we compare structures refined without QM, as well as with QM with the optimum weights *w*_N_ = 1 and *w*_X_ = 3. It can be seen that the largest movements are observed for the D atoms, in particular those of water molecules, whereas the heavy atoms typically remain at the same positions.

**Figure 6:**
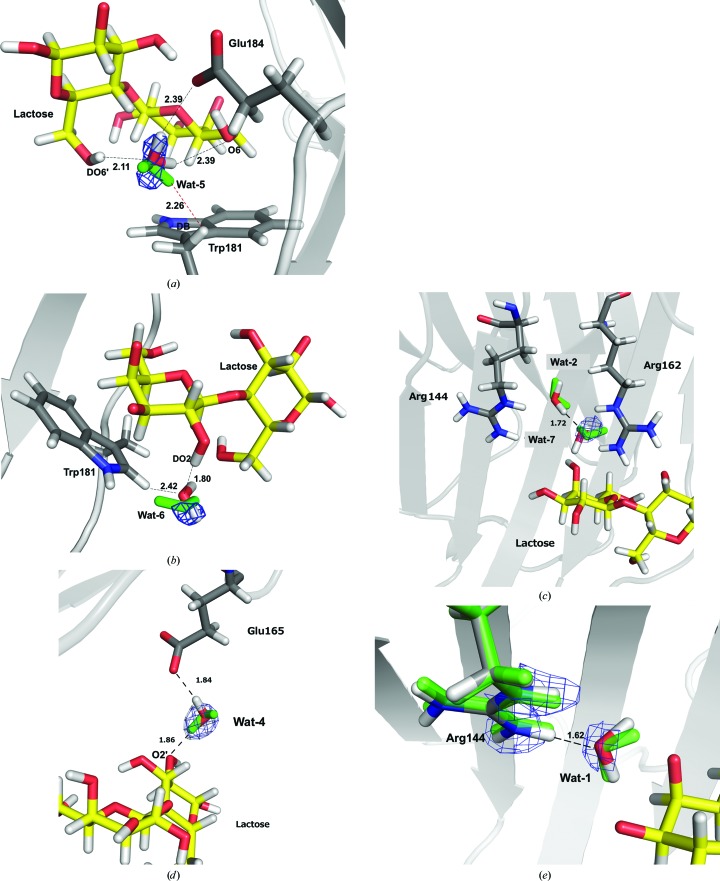
Significant movement of four water molecules and one protein residue in the QM refinement of the preliminary neutron structure of galectin-3: Wat-5 (*a*), Wat-6 (*b*), Wat-7 (*c*), Wat-4 (*d*) and Arg144 (*e*). Atoms are shown in green before and red/white after QM refinement. The nuclear 2*m*|*F*_o_| − *D*|*F*_c_| density is shown in light blue at 1.0σ and in dark blue at 0.7σ (for the less well ordered water molecules Wat-5 and Wat-6).

For example, one of the largest changes is observed for the weakly bound water molecule 5, which shows quite unusual hydrogen bonds in the original structure, with one D atom directed towards the side-chain DB atom of Trp181 (2.26 Å) and the other D atom pointing towards the solvent (Fig. 6 ▸ *a*). In the QM-refined structure, this water molecule rotates by 90° so that the deuterons are directed towards the O6 atom of lactose (2.39 Å) and one of the OE atoms of Glu184 (2.39 Å). In both cases, the water molecule also receives a hydrogen bond from the DO6′ atom of lactose (2.11 and 2.27 Å, respectively).

Wat-6 is also weakly bound and shows large changes in the positions of the D atoms (Fig. 6 ▸ *b*). In the original structure it does not form any hydrogen bonds. Instead, one of the deuterons is only 2.48 Å from DE1 of Trp181, in a seemingly unfavourable interaction. In the QM-refined structures Wat-6 rotates so that it donates a hydrogen bond to O6′ of lactose. In both cases, it also receives a hydrogen bond from the DO2 atom of lactose, but this interaction is also significantly improved in the QM-refined structure, with a D–O distance of 1.80 Å compared with 2.06 Å.

Wat-7 also shows a rather large reorganization (Fig. 6 ▸ *c*). In the original structure one of the D atoms points towards the D atom of another water molecule (Wat-2, 1.69 Å). In the QM-refined structure this D atom instead points towards lactose (but without forming any particular hydrogen bond) so that the O atom can receive a strong hydrogen bond from Wat-2 (D–O distance of 1.72 Å).

Finally, Wat-4 also experiences a reorganization of the hydrogen-bond network (Fig. 6 ▸ *d*). In the original structure one of the D atoms points towards the solution. However, in the QM-refined structure it instead forms a strong hydrogen bond to the OE2 atom of Glu-165 (1.84 Å). In both cases the other D atom forms a hydrogen bond to O2′ of lactose, although it is shortened by 0.1 Å in the QM/MM structure (from 1.99 to 1.86 Å). Thereby, it bridges the interaction between Glu-165 and the ligand.

In the ligand, the DO1′ atom shows the largest movement, with an ∼100° change in the DO1′—O1′—C1′—C2′ dihedral angle (Fig. 5 ▸). This is probably to relieve a short contact between DO1′ and DO2′ (2.05 Å). However, in neither the original structure nor in the QM-refined structure does DO1′ form any hydrogen bond to the surroundings (it is solvent-exposed, but there are no visible water molecules close to it). The movement of DO2′ is negligible.

The other atoms in both the ligand and the protein show only smaller adjustments within the same local minimum, typically to improve hydrogen-bond interactions. For example, DO4 in lactose moves by 0.70 Å to shorten the hydrogen-bond distance to NE2 of His158 from 2.16 to 1.68 Å (Fig. 5 ▸). The largest movement of a protein atom is that of DH12 of Arg144, which moves to improve the hydrogen bond to a water molecule (Wat-1) from 1.77 to 1.62 Å, as shown in Fig. 6 ▸(*e*). On average, the protein atoms in the QM system move by only 0.06–0.17 Å, again with the largest movement for Arg144.

**Figure 7:**
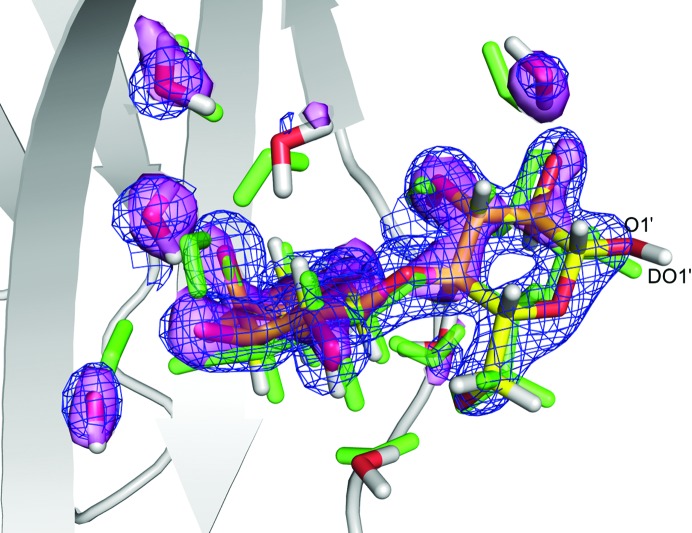
Positions of lactose and the water molecules in QM/MM structures optimized without (*w*_X_ = *w*_N_ = 0, green) or with restraints to the crystallographic data (*w*_N_ = 1, *w*_X_ = 3). The 2*m*|*F*_o_| − *D*|*F*_c_| electron density at 1.0σ is shown as a blue grid and the nuclear 2*m*|*F*_o_| − *D*|*F*_c_| density at 1.0σ is shown as a violet surface.

If we instead compare the best QM-refined structure with a pure QM/MM structure, obtained with *w*_N_ = *w*_X_ = 0, much larger movements are observed, as can be seen in Fig. 7 ▸. In fact, the median movement of the atoms in the QM system is 0.5 Å, compared with 0.1 Å for the QM-refined structure. For most residues this represents a slight change (rotation or tilt) in the orientation of the amino-acid side chains. However, several water molecules move by up to 1.8 Å and several lactose atoms also move significantly (up to 1.7 Å for O1′ and DO1′). This larger movement of course reflects that lactose and water are not as restrained by the surrounding structure as the amino-acid side chains, which are kept in place by the backbone atoms (which were kept fixed in the optimization). However, as can be seen from the figure, the water molecules move away from the electron and neutron densities, illustrating the advantage of the joint QM refinement. The discrepancy between the QM/MM and experimental data is caused by the fact that the latter include dynamics and entropy, as well as the averaged effects of the surrounding solvent, but also disorder in the crystal.

**Figure 8:**
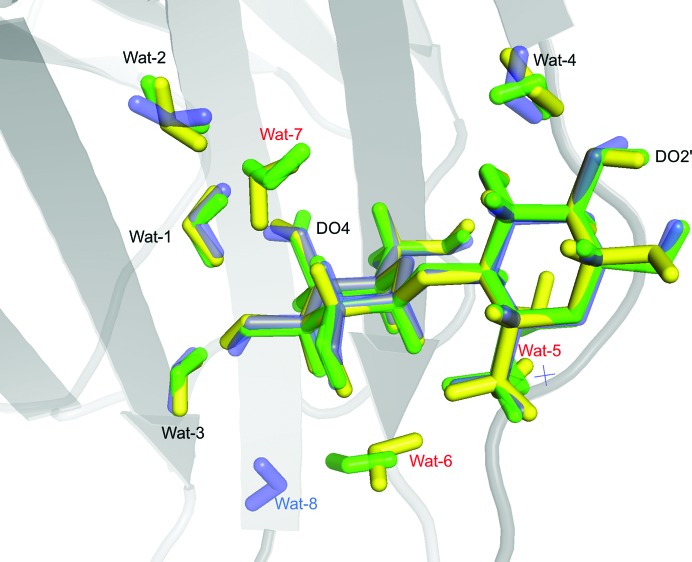
Overlay of the deposited galectin-3 structure (PDB entry 6eym, blue; Manzoni *et al.*, 2018 ▸) and the preliminary structure before (green) and after (yellow) QM refinement. Protein residues are hidden for clarity.

Next, we studied the deposited neutron structure of lactose bound to galectin 3 (PDB entry 6eym), which has a higher completeness of the neutron data and was previously joint-refined in *PHENIX* 1.12. Compared with the preliminary structure, the deposited structure contains fewer water molecules around the lactose molecule (Fig. 8 ▸). Interestingly, three of the four water molecules (Wat-5, Wat-6 and Wat-7) that showed large reorganization after quantum refinement are absent in the more conservatively interpreted deposited structure, suggesting that these water molecules were not well ordered in the original structure. Nuclear difference density for these waters can be seen in PDB entry 6eym at low contour levels. Moreover, Wat-4 in the deposited structure is in the orientation suggested by quantum refinement of the first structure, proving that QM refinement of the preliminary structure found the correct orientation of this water molecule.

We also performed quantum refinement of the deposited structure, including only five water molecules in the QM system: Wat-1, Wat-2, Wat-3, Wat-4 and Wat-8 (see Table 1 ▸ for the corresponding water numbers in the deposited structure, PDB entry 6eym). Wat-8, which was not present in the preliminary structure, forms hydrogen bonds to Wat-3 and lies close to Trp181. The lactose molecule also shows a different orientation of two D atoms, DO4 and DO2′. Strikingly, in the deposited structure the DO4 atom is close to the position in the quantum refinement of the preliminary structure. No protein residues show any significant reorientation between the two structures.

**Figure 9:**
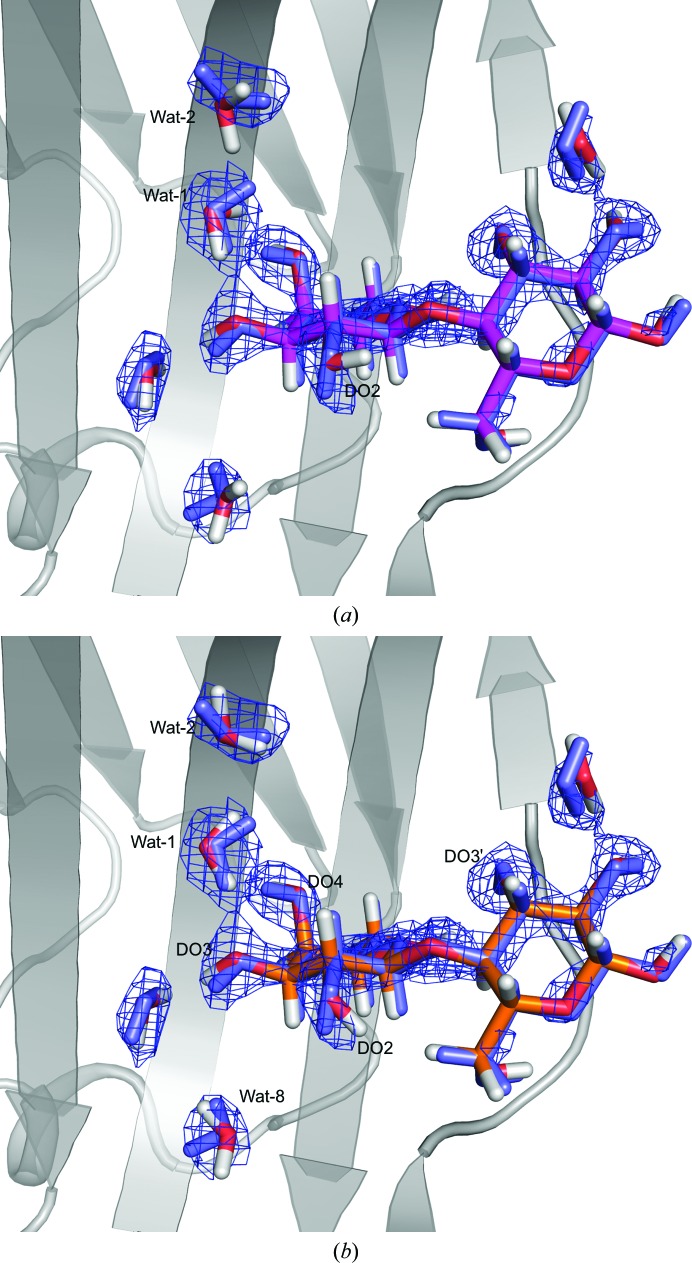
Overlay of the deposited galectin-3 structure before (blue) and after QM refinement with (*a*) *w*_X_ = 3 and *w*_N_ = 1 (C atoms in magenta) and (*b*) *w*_X_ = *w*_N_ = 7 (C atoms in orange). The nuclear 2*m*|*F*_o_| − *D*|*F*_c_| density at 0.8σ is shown in blue. Protein residues are hidden for clarity.

The joint QM refinement of the deposited structure, using the optimized weights from the previous QM refinement, *w*_N_ = 1 and *w*_X_ = 3, gave an *R*_free_ of 0.211 for the neutron data and 0.156 for the X-ray data (after bulk-solvent correction and *B*-factor refinement in *PHENIX*). However, with these weights several atoms move out of the nuclear density after quantum refinement, for example Wat-1, Wat-2 and the ligand DO2 atom (Fig. 9 ▸ *a*). Therefore, we performed two sets of weight searches starting from two relative weights: *w*_X_/*w*_N_ = 3, which were the weights used in the preliminary structure, and *w*_X_/*w*_N_ = 1 as used in Manzoni *et al.* (2018 ▸). We used a linear search from *w*_N_ = *w*_X_ = 1 to *w*_N_ = *w*_X_ = 10 and from *w*_N_ = 1 and *w*_X_ = 3 to *w*_N_ = 10 and *w*_X_ = 30, respectively. In order to compare the quality of the resulting structures, we calculated the maximum absolute RSZD values of lactose and water molecules from the neutron maps, which measure the local accuracy of the model, being sensitive to local discrepancies. As can be seen in Table 3 ▸, the structure obtained from QM refinement with *w*_N_ = *w*_X_ = 7 fits the crystallographic data best, with the RSZDs of Wat-1 and the lactose molecule being over one unit lower than in QM refinements using lower weights. However, other weights can be used to obtain structures of similar quality, for example structures obtained with *w*_N_ = *w*_X_ = 6 or *w*_N_ = 5 and *w*_X_ = 15 give rise to similar RSZD values of the residues in the quantum system. The *R*_free_ values of the resulting structures do not change compared with those obtained from the QM refinement with *w*_N_ = 1 and *w*_X_ = 3 (0.213 and 0.159; Table 2 ▸). For the higher values of the two weights the improvement in the RSZD scores is small and for weights of higher than 10 convergence problems appeared in the QM calculations.

Fig. 9 ▸(*b*) shows a comparison between the original deposited structure and the QM-refined structure with *w*_N_ = *w*_X_ = 7 (virtually identical results were also obtained with *w*_N_ = 5 and *w*_X_ = 15). As in the QM refinement of the preliminary structure, the largest movements are observed for D atoms, although to a smaller degree, as the neutron data are of higher quality than in the preliminary structure. The water molecules still change their orientation to form more favourable hydrogen bonds, suggesting that QM refinement can also improve structures of high quality.

**Figure 10:**
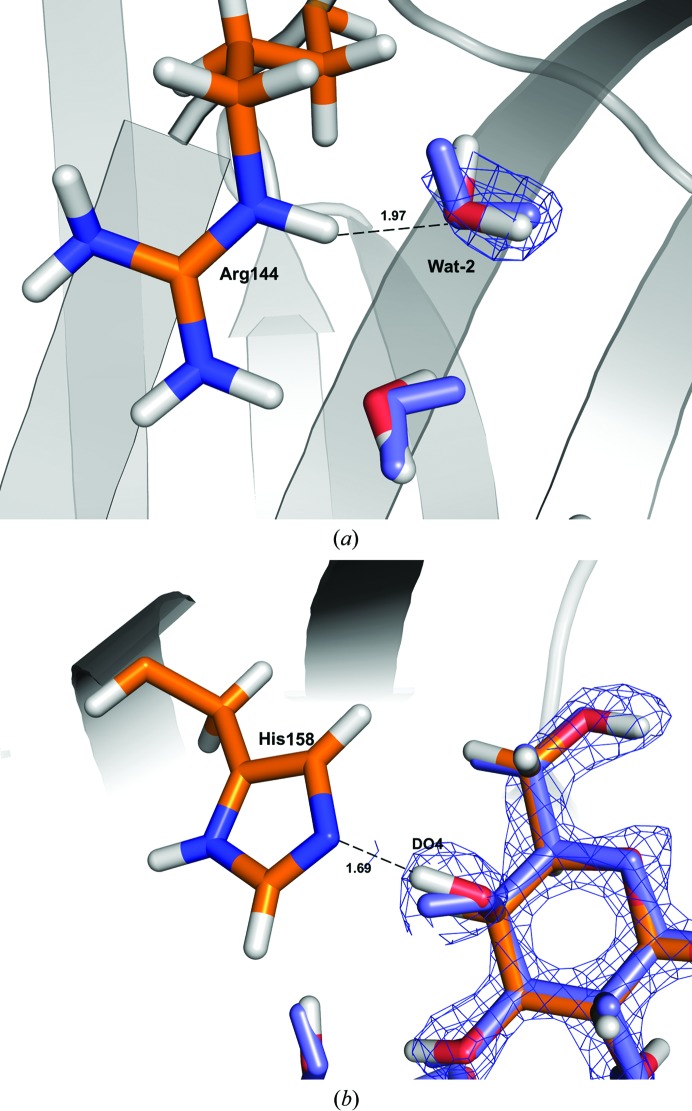
Movement of water molecule Wat2 (*a*) and of the DO4 atom in the lactose molecule (*b*) in order to form more favourable hydrogen bonds after QM refinement of the deposited structure with *w*_X_ = *w*_N_ = 7. The nuclear 2*m*|*F*_o_| − *D*|*F*_c_| density at 1.0σ is shown in blue.

One such movement is observed for Wat-2. The QM refinement orients its O atom to form a hydrogen bond to Arg-144 (Fig. 10 ▸ *a*), as in the QM refinement of the preliminary structure. The new water molecule introduced into the QM system (Wat-8) also rotates slightly so that one D atom now points towards solution.

In the ligand, several D atoms show small adjustments to form more favourable hydrogen bonds: DO3 to Wat-3, DO4 to His158 and DO3′ to Glu184. The QM refinement is particularly important for the conformation of the DO4 atom, as the nuclear density does not permit an unambiguous assignment of its position (Fig. 10 ▸ *b*). DO2 shows the largest movement, but it does not form a hydrogen bond, as the water molecule that it was hydrogen-bonded to in the preliminary structure (Wat-6) is not present in the deposited structure. The protein atoms do not show any significant changes in their position, as in the first QM refinement.

To check that the QM refinement had not deteriorated the protein structure, validation statistics were calculated with *MolProbity* for the final structures (*w*_N_ = 1 and *w*_X_ = 3 for the preliminary structure and *w*_N_ = *w*_X_ = 7 for the deposited structure). As can be seen in Table 2 ▸, the structures show the same quality as before QM refinement, whereas the *R*/*R*_free_ values show a small improvement when compared with the *R*/*R*_free_ values obtained from the standard re-refinement performed in *nCNS*.

Finally, we checked that the results are not sensitive to the starting coordinates by randomly modifying the coordinates by up to 0.1 Å (using *phenix.pdbtools*) in the QM system and reperforming the QM refinement. This led to the same optimum structure (r.m.s.d. difference of the QM system of 0.05 Å). Therefore, the water movements reported in this article are outside the statistical uncertainty of the results.

### 3.3. Application to lytic polysaccharide monooxygenase (LPMO)   

The primary use of quantum refinement is for systems for which the standard chemical restraints can be expected to be poor (for example metal sites or nonstandard ligands or inhibitors) or where standard refinement gives ambiguous results. To illustrate such a typical application, we have also tested the method on the neutron structure of a copper-containing metalloenzyme, LPMO. In a recent study, Bacik and coworkers suggested that in subunit *B* of the enzyme the terminal N atom, which coordinates the Cu atom, is partially deprotonated (Bacik *et al.*, 2017 ▸). This was supported by the presence of an asymmetric nuclear difference-density peak around the N-terminal atom. However, the interpretation is rather ambiguous, as the model was only refined against neutron data at 2.1 Å resolution and the N-terminal deuterons were not modelled explicitly in the structure. Therefore, in order to discern whether a protonated or deprotonated N-terminus fits the data better, we performed joint QM refinement against both X-ray and neutron data. This is suitable as the space groups and unit-cell parameters of both the X-ray and neutron crystals are nearly identical.

To determine the optimum weights, we performed a similar linear search to that performed for the deposited galectin structure. We kept the relative weight factors with *w*_X_/*w*_N_ = 1 (which are the default in *phenix.refine*) and increased them stepwise from *w*_X_ = 1 to *w*_X_ = 10. We then compared the RSZD scores of the residues in the quantum system (including the Cu atom and the peroxide molecule). All calculations were performed on subunit *A*, as this showed no ambiguity in the protonation of the N-terminus. As can be seen in Table 4 ▸, different weights did not change the fit of the structure to the data, with all RSZD scores being almost identical for the various weights. This is not completely surprising, as the QM system is quite rigid and the data quality is worse than for the galectin structure. However, if the weights are further decreased (*w*_N_ = *w*_X_ = 0.1 or smaller) the RSZD increases significantly, showing that the structure becomes too biased towards QM.

**Figure 11:**
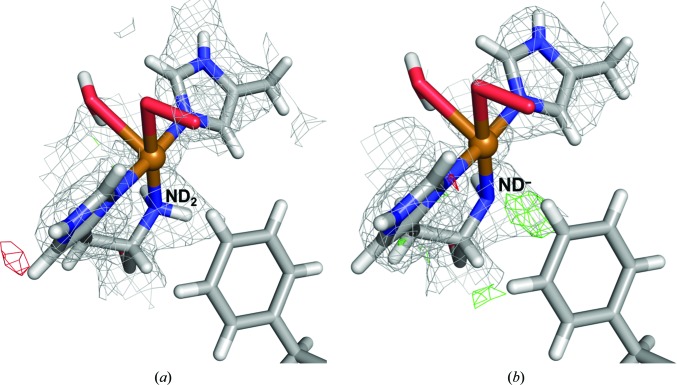
Structure and nuclear density maps of the active site of LPMO in subunit *B* after QM refinement. (*a*) The N-terminus in the protonated –ND_2_ form and (*b*) the N-terminus in the deprotonated ND^–^ form. The 2*m*|*F*_o_| − *D*|*F*_c_| nuclear density at 1.0σ is shown as a blue grid and *m*|*F*_o_| − *D*|*F*_c_| nuclear difference density is shown at 3.0σ (green grid) and −3.0σ (red grid).

Therefore, we selected *w*_N_ = *w*_X_ = 1 (which gave the lowest RSZD scores and will not bias the structure towards either the QM or the crystallographic data) and performed a QM refinement with subunit *B* in the quantum system. We compared the results of using two different models, one with the N-terminus protonated (*i.e.* modelled as –ND_2_) and the other deprotonated (*i.e.* modelled as –ND^–^). Fig. 11 ▸ clearly shows a positive difference density close to the N-terminus in the deprotonated model (green grid in the centre of Fig. 11 ▸ *b*), indicating that modelling the N-terminus as a protonated –ND_2_ group is a better interpretation of the joint neutron and X-ray data. A more in-depth discussion of these calculations and their implication for the biological function of the enzyme has been published elsewhere (Caldararu *et al.*, 2018 ▸).

The refinement statistics for the final QM-refined structure are shown in Table 5 ▸. As for the galectin structure, the overall structure of the protein keeps its integrity and the *R*/*R*_free_ values are slightly improved compared with those calculated with *nCNS* before the QM refinement.

## 4. Conclusions   

In this study, we have implemented the combination of joint neutron and X-ray crystallographic refinement with QM/MM calculations. We have combined the freely available *nCNS* software (Adams *et al.*, 2009 ▸) with the QM software *TURBOMOLE* (Furche *et al.*, 2014 ▸). We have applied the method to a recent neutron structure of lactose bound to the carbohydrate-recognition domain of galectin-3 (Manzoni *et al.*, 2018 ▸), and to the active site of LPMO (Caldararu *et al.*, 2018 ▸). We show that the method behaves properly and that we can use the two weight factors *w*_N_ and *w*_X_ to bias the structure towards the neutron data, the X-ray data or the QM structure. We can obtain an ideal compromise between the QM and the crystallographic methods by monitoring the RSZD values of residues in the QM system (see Tables 3 ▸ and 4 ▸), whereas optimizing the crystallo­graphic *R*_free_ values seems to give less reliable results.

The approach allows us to identify and remove problems in the corresponding structure optimized with conventional methods without the use of QM. In particular, the description of the hydrogen-bonding pattern is improved, with a level of detail and confidence that is not possible with standard refinement. For the solvent-accessible binding site in galectin-3, it is primarily the solvent molecules and the D atoms of the ligand that are improved by QM refinement. In several cases, the hydrogen-bonding pattern was modified to avoid steric clashes or to improve the hydrogen-bond strengths. For LPMO, we show that the method can be used to determine the protonation states of interesting residues by comparing the structures refined in the different states. Of course, the improvements are most important when the experimental data are poorer, as the QM refinements of the preliminary structures of galectin-3 and of LPMO showed.

There is no significant difference in either the *R* values or the density maps between the normal and QM-refined structures, which means that both models essentially fit the crystallographic data equally well. However, the QM-refined models include more sophisticated chemical information and hence allow more confidence in drawing functional or mechanistic conclusions from the structure.

Naturally, this improvement comes with an additional cost. The QM refinements took around 5 h each, whereas standard neutron/X-ray refinement takes only a few minutes (both on a single processor). On the other hand, the QM system was quite large for the solvent-exposed site of galectin-3 (153 atoms). However, the method is intended to be used primarily at the end of the refinement procedure and only for sites of particular interest, as shown in the application to LPMO. Moreover, some hours of calculation are not much compared with the time required to grow the large crystals that are needed for neutron crystallography or even with the data-collection time.

A limitation of the present QM refinement approach is that it can only model a single conformation of the atoms in the QM system. This means that if a D atom or a water molecule may form several different hydrogen bonds of similar stability, the QM refinement will probably converge to one of these, typically that favoured by enthalpic factors and by the QM hydrogen-bond energy, unless the density maps show that the D atom is clearly in between. Naturally, this problem can be solved by simply using two separate QM calculations, one for each conformation, but this would double the computational cost and give an exponential increase in the number of QM systems if there is more than one alternative conformation.

A related problem is that the QM-refined structures will be biased towards the formation of hydrogen bonds within the QM system. The reason for this is that there are no electrostatic interactions with the MM surroundings in the current implementation (following the convention of neutron and X-ray crystallography). The user needs to remember this problem when interpreting the structures. Technically, it is trivial to turn on the electrostatics in the calculations. However, this would introduce several conceptual problems. Firstly, the calculations will no longer be directly comparable with standard refinement, making the comparison more complicated. Secondly, all D atoms are not unambiguously discernible in the neutron structure. However, the electrostatics strongly depend on the position of these atoms, at least the polar D atoms. This makes it necessary to guess the positions of all D atoms. This can in principle be performed with standard MM software. However, it means that the final structure will be a mixture of observed and speculated atomic positions. Thirdly, alternative conformations will again be a problem, because the QM atoms can only interact with one set of charges. For these reasons, we have decided to not include electrostatics in this first version of the QM refinement procedure.

## Supplementary Material

R factors for different values of the weights; topology and parameter files for lactose; coordinates for the re-refined structures.. DOI: 10.1107/S205979831900175X/ei5038sup1.pdf

## Figures and Tables

**Table 1:** Correspondence between the water molecules discussed in the text and their numbers in the deposited structure (PDB entry 6eym)

Text	PDB entry 6eym
Wat-1	Wat-648
Wat-2	Wat-668
Wat-3	Wat-666
Wat-4	Wat-614
Wat-5	—
Wat-6	—
Wat-7	—
Wat-8	Wat-655

**Table 2:** Structure quality of the two galectin-3 structures before and after QM refinement, with *w*_N_ = 1 and *w*_X_ = 3 for the preliminary structure and *w*_N_ = 7 and *w*_X_ = 7 for the deposited structure *R*_work_ and *R*_free_ were calculated in *nCNS*. Geometry statistics were calculated with *MolProbity*.

	Preliminary		Deposited	
	Before	After	Before	After
*R*_work_ (X-ray)	0.181	0.179	0.189	0.189
*R*_free_ (X-ray)	0.208	0.207	0.195	0.195
*R*_work_ (neutron)	0.221	0.216	0.219	0.218
*R*_free_ (neutron)	0.238	0.233	0.221	0.221
R.m.s.d., bonds (Å)	0.021	0.022	0.011	0.018
R.m.s.d., angles (°)	1.9	1.9	1.7	1.7
Ramachandran favoured (%)	98.5	98.5	97.1	98.5
Ramachandran allowed (%)	3.3	1.5	2.9	1.5
Ramachandran outliers (%)	0.0	0.0	0.0	0.0
Rotamer outliers (%)	0.8	0.8	0.0	0.0
All-atom clashscore	4.87	3.10	4.00	1.77

**Table 3:** Maximum absolute RSZD of the lactose molecule and of the five water molecules included in the QM refinement of the deposited galectin-3 structure obtained with different weights of the experimental data (*w*_N_ and *w*_X_) Note that *w*_X_/*w*_N_ = 1 (left part of table) or 3 (right part).

*w*_N_	1	2	3	4	5	6	7	8	9	10		1	2	5	10
*w*_X_	1	2	3	4	5	6	7	8	9	10		3	6	15	30
Lactose	0.6	0.8	0.8	0.4	0.3	0.3	0.2	0.8	0.2	2.8		1.5	1.6	0.2	0.2
Wat-1	1.5	1.5	1.2	0.6	0.4	0.1	0.0	1.6	0.1	0.1		1.4	1.1	0.1	0.1
Wat-2	0.9	0.9	0.9	0.6	0.8	0.7	0.7	0.9	0.9	1.0		1.0	0.9	1.0	1.4
Wat-3	1.2	0.8	0.8	0.9	0.8	0.8	0.8	0.9	0.8	0.7		0.9	1.0	0.9	0.8
Wat-4	1.9	1.6	1.5	1.3	1.1	0.9	0.8	1.6	0.6	0.6		1.1	0.9	0.6	0.5
Wat-8	0.8	0.8	0.8	0.6	0.6	0.8	0.9	0.7	1.1	1.1		0.5	0.5	0.9	0.9
**Sum**	**6.9**	**6.4**	**6.0**	**4.4**	**4.0**	**3.6**	**3.4**	**6.5**	**3.7**	**6.3**		**6.4**	**6.0**	**3.7**	**3.9**

**Table 4:** Maximum absolute RSZD of the residues in the QM system in the QM refinement of subunit *A* of the LPMO structure obtained with different weights of the experimental data (*w*_N_ and *w*_X_) Note that *w*_X_/*w*_N_ = 1 in all refinements.

*w*_N_	0.001	0.01	0.1	1	2	3	4	5	6	7	8	9	10
*w*_X_	0.001	0.01	0.1	1	2	3	4	5	6	7	8	9	10
Copper	1.2	1.2	1.2	1.2	1.2	1.3	1.2	1.2	1.2	1.2	1.2	1.2	1.2
Peroxide	0.1	0.1	0.1	0.1	0.1	0.1	0.1	0.1	0.1	0.1	0.1	0.1	0.1
His32	2.5	2.4	2.1	0.7	0.7	0.7	0.7	0.8	0.8	0.8	0.8	0.8	0.8
His109	1.0	1.0	0.9	0.2	0.2	0.2	0.3	0.3	0.3	0.3	0.3	0.3	0.3
Phe164	0.5	0.5	0.5	0.5	0.5	0.5	0.5	0.5	0.5	0.5	0.5	0.5	0.5
Wat-301	0.9	0.9	0.9	0.9	0.9	0.9	0.9	0.9	0.9	0.9	0.9	0.9	0.9
**Sum**	**6.2**	**6.1**	**5.7**	**3.6**	**3.6**	**3.7**	**3.7**	**3.8**	**3.8**	**3.8**	**3.8**	**3.8**	**3.8**

**Table 5:** Structure quality of the LPMO structure before and after QM refinement with the active site in subunit *B* with two deuterons on the N-terminus as the QM system *R*_work_ and *R*_free_ were calculated in *nCNS*. Geometry statistics were calculated with *MolProbity*.

	Before	After
*R*_work_ (X-ray)	0.161	0.150
*R*_free_ (X-ray)	0.164	0.153
*R*_work_ (neutron)	0.243	0.237
*R*_free_ (neutron)	0.248	0.244
R.m.s.d., bonds (Å)	0.009	0.008
R.m.s.d., angles (°)	0.9	0.9
Ramachandran favoured (%)	98.5	99.2
Ramachandran allowed (%)	1.5	0.8
Ramachandran outliers (%)	0.0	0.0
Rotamer outliers (%)	0.0	0.0
All-atom clashscore	1.82	2.93
