# Refinement of protein structures using a combination of quantum-mechanical calculations with neutron and X-ray crystallographic data. Corrigendum

**DOI:** 10.1107/S2059798319016383

**Published:** 2020-01-01

**Authors:** Octav Caldararu, Francesco Manzoni, Esko Oksanen, Derek T. Logan, Ulf Ryde

**Affiliations:** a Division of Theoretical Chemistry, Lund University, Chemical Centre, PO Box 124, SE-221 00, Lund, Sweden; b Department of Biochemistry and Structural Biology, Lund University, Chemical Centre, PO Box 124, SE-221 00, Lund, Sweden; c European Spallation Source ESS ERIC, PO Box 176, SE-221 00, Lund, Sweden

**Keywords:** refinement of protein structures, quantum-mechanical calculations, neutrons, corrigendum

## Abstract

Corrections are published for the article by Caldararu *et al.* [(2019), *Acta Cryst.* D**75**, 368–380].

## Abstract 2

The article by Caldararu *et al.* [(2019), *Acta Cryst.* D**75**, 368–380] is corrected.

In our previous article, *Refinement of protein structures using a combination of quantum-mechanical calculations with neutron and X-ray crystallographic data* (Caldararu, Manzoni *et al.*, 2019 ▸), there was a small technical error in calculating the nuclear scattering-length density maps for Fig. 11 and Table 4.

Joint X-ray–neutron quantum refinement of lytic polysaccharide monooxygenase (LPMO) was conducted even though the unit-cell parameters of the X-ray crystal and of the neutron crystal are not exactly equal. The quantum refinement calculations were performed in the X-ray unit cell, but the nuclear density maps in Fig. 11 were calculated in the neutron unit cell. We report below the corrected figure (Fig. 1 ▸), with nuclear density maps calculated in the X-ray unit cell. The RSZD (real-space difference-density *Z*) values used in weight determination for the LPMO quantum refinement in the original article were also calculated from the maps in the wrong unit cell. A corrected table with the maximum absolute RSZD of the residues of the active site is shown below (Table 1 ▸). It can be seen that the absolute values of the RSZD scores have changed somewhat (by up to 1.2), but the variation with the X-ray and neutron weight factors, *w*_N_ and *w*_X_, does not change much. All RSZD scores are almost constant for *w*_N_ = *w*_X_ = 1–10, whereas RSZD increases if the weights are further decreased. Therefore, the selection of *w*_N_ = *w*_X_ = 1 as proper weights is still in accordance with the RSZD values estimated from maps calculated in the correct unit cell.

In the corrected version of the figure, there is no positive difference density at the deprotonated N-terminus at the 3.0σ level. However, there is still positive difference density around the N-terminus at the 2.8σ level as can be seen in the corrected Fig. 1 ▸(*b*). This level is still well above the noise level of the nuclear density maps in that area (∼2.3σ). Although the maps without the D2 atom at the N-terminus look more similar to the ones reported by Bacik *et al.* (Bacik *et al.*, 2017 ▸), the structures with two deuterium atoms still fit better to the neutron data. Thus, our conclusions about the use of quantum refinement in elucidating protonation states in metallo­enzymes do not change.

Most importantly, the correction in no way affects the combined quantum mechanical and joint X-ray–neutron refinement method, which was presented in the article. The LPMO quantum refinement was included only as a second application of the method, taken from another publication (Caldararu, Oksanen *et al.*, 2019*a* ▸), for which a more detailed correction has already been submitted (Caldararu, Oksanen *et al.*, 2019*b* ▸).

## Figures and Tables

**Figure 1:**
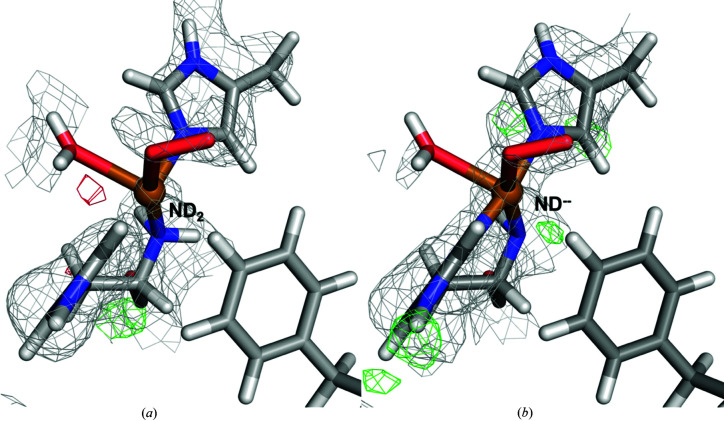
Structure and nuclear density maps of the active site of LPMO in subunit *B* after QM refinement. (*a*) The N-terminus in the protonated –ND_2_ form and (*b*) the N-terminus in the deprotonated ND^–^ form. The 2*m*|*F*_o_| − *D*|*F*_c_| nuclear density at 1.0σ is shown as a grey grid and *m*|*F*_o_| − *D*|*F*_c_| nuclear difference density is shown at 2.8σ (green grid) and −2.8σ (red grid). This is a corrected version of Fig. 11 from Caldararu, Manzoni *et al.* (2019 ▸).

**Table 1:** Maximum absolute RSZD values of the residues in the QM system in the QM refinement of subunit *A* of the LPMO structure obtained with different weights of the experimental data (*w*_N_ and *w*_X_) This is a corrected version of Table 4 from Caldararu, Manzoni *et al.* (2019 ▸). Note that *w*_X_/*w*_N_ = 1 in all refinements.

*w*_N_	0.001	0.01	0.1	1	2	3	4	5	6	7	8	9	10
*w*_X_	0.001	0.01	0.1	1	2	3	4	5	6	7	8	9	10
Cu	0.4	0.4	0.4	0.4	0.4	0.4	0.4	0.4	0.4	0.4	0.4	0.4	0.4
Peroxide	0.3	0.3	0.1	0.1	0.1	0.0	0.0	0.0	0.0	0.0	0.0	0.0	0.0
His32	1.7	1.5	0.9	0.4	0.4	0.4	0.4	0.4	0.4	0.4	0.4	0.4	0.4
His109	1.0	1.0	0.8	0.7	0.7	0.7	0.7	0.7	0.7	0.7	0.7	0.7	0.7
Phe164	1.4	1.4	1.4	1.4	1.4	1.4	1.4	1.4	1.4	1.4	1.4	1.4	1.4
Wat301	1.7	1.7	1.8	1.7	1.7	1.7	1.7	1.7	1.7	1.7	1.7	1.7	1.7
**Sum**	**6.5**	**6.3**	**5.5**	**4.7**	**4.7**	**4.6**	**4.6**	**4.6**	**4.6**	**4.6**	**4.6**	**4.6**	**4.6**
