# Supplementary material for: Refinement of protein structures using a combination of quantum-mechanical calculations with neutron and X-ray crystallographic data
Source: Acta Crystallogr D Struct Biol. 2019 Mar 28;75(Pt 4):368–80. doi: 10.1107/S205979831900175X (PMC6465982; doi:10.1107/S205979831900175X)
Supplement: Supplementary file 1 [file d-75-00368-sup1.pdf]

STRUCTURAL BIOLOGY

Volume 75 (2019)

Supporting information for article:

Refinement of protein structures using a combination of quantum-mechanical calculations with neutron and X-ray crystallographic data

Octav Caldararu, Francesco Manzoni, Esko Oksanen, Derek T. Logan and Ulf Ryde

Table S1. *R* factors obtained from the refinement with different values of w_N and w_X.

w_N	w_X	R^N_{free}	R^N	R^N_{free}	R^X
0.00	0.00	0.2467	0.2284	0.2121	0.1927
0.00	0.01	0.2459	0.2259	0.2108	0.1899
0.00	0.03	0.2447	0.2240	0.2099	0.1878
0.00	0.10	0.2406	0.2204	0.2087	0.1842
0.00	0.30	0.2364	0.2181	0.2080	0.1817
0.00	1.00	0.2333	0.2173	0.2077	0.1803
0.00	3.00	0.2332	0.2167	0.2069	0.1796
0.00	10.00	0.2335	0.2165	0.2065	0.1791
0.01	0.00	0.2461	0.2264	0.2111	0.1906
0.01	0.01	0.2454	0.2251	0.2105	0.1892
0.01	0.03	0.2441	0.2234	0.2097	0.1872
0.01	0.10	0.2402	0.2202	0.2086	0.1840
0.01	0.30	0.2364	0.2181	0.2079	0.1817
0.01	1.00	0.2334	0.2173	0.2077	0.1803
0.01	3.00	0.2331	0.2167	0.2069	0.1796
0.01	10.00	0.2336	0.2165	0.2066	0.1791
0.03	0.00	0.2454	0.2247	0.2103	0.1890
0.03	0.01	0.2446	0.2237	0.2100	0.1879
0.03	0.03	0.2434	0.2225	0.2093	0.1864
0.03	0.10	0.2399	0.2197	0.2085	0.1836
0.03	0.30	0.2354	0.2182	0.2077	0.1816
0.03	1.00	0.2334	0.2172	0.2077	0.1803
0.03	3.00	0.2332	0.2167	0.2070	0.1796
0.03	10.00	0.2337	0.2165	0.2065	0.1791
0.10	0.00	0.2424	0.2210	0.2094	0.1856
0.10	0.01	0.2419	0.2208	0.2091	0.1853
0.10	0.03	0.2408	0.2201	0.2087	0.1844
0.10	0.10	0.2384	0.2188	0.2081	0.1829
0.10	0.30	0.2349	0.2179	0.2078	0.1815
0.10	1.00	0.2334	0.2171	0.2077	0.1802
0.10	3.00	0.2332	0.2166	0.2070	0.1796
0.10	10.00	0.2337	0.2165	0.2066	0.1791
0.30	0.00	0.2379	0.2183	0.2083	0.1833
0.30	0.01	0.2377	0.2183	0.2082	0.1831
0.30	0.03	0.2373	0.2181	0.2081	0.1829
0.30	0.10	0.2364	0.2178	0.2078	0.1822
0.30	0.30	0.2343	0.2174	0.2078	0.1812
0.30	1.00	0.2333	0.2168	0.2076	0.1801
0.30	3.00	0.2332	0.2164	0.2068	0.1796
0.30	10.00	0.2337	0.2164	0.2066	0.1791
1.00	0.00	0.2347	0.2165	0.2077	0.1817
1.00	0.01	0.2349	0.2166	0.2078	0.1817
1.00	0.03	0.2353	0.2167	0.2079	0.1816
1.00	0.10	0.2351	0.2166	0.2080	0.1813
1.00	0.30	0.2341	0.2164	0.2080	0.1807
1.00	1.00	0.2336	0.2162	0.2073	0.1800
1.00	3.00	0.2332	0.2160	0.2068	0.1795
1.00	10.00	0.2330	0.2159	0.2060	0.1807
3.00	0.00	0.2343	0.2154	0.2074	0.1810
3.00	0.01	0.2343	0.2154	0.2074	0.1810
3.00	0.03	0.2343	0.2153	0.2074	0.1810
3.00	0.10	0.2343	0.2154	0.2074	0.1808
3.00	0.30	0.2344	0.2153	0.2073	0.1805
3.00	1.00	0.2343	0.2154	0.2075	0.1800
3.00	3.00	0.2338	0.2154	0.2070	0.1795
3.00	10.00	0.2341	0.2156	0.2069	0.1791
10.00	0.00	0.2347	0.2144	0.2070	0.1813
10.00	0.01	0.2347	0.2145	0.2071	0.1813
10.00	0.03	0.2347	0.2144	0.2071	0.1812
10.00	0.10	0.2347	0.2145	0.2071	0.1811
10.00	0.30	0.2347	0.2144	0.2072	0.1808
10.00	1.00	0.2345	0.2145	0.2073	0.1803
10.00	3.00	0.2344	0.2146	0.2075	0.1797
10.00	10.00	0.2343	0.2149	0.2073	0.1791

Table S2. The CNS topology file employed for lactose.

```
remark Topology file for LAT, UR & FM 27/1-15
set echo=false end
autogenerate angles=true end
{}
mass    CH1E  12.011
mass    HA     1.008
mass    CH2E  12.011
mass    OS    15.999
mass    OH1   15.999
mass    D      1.008
{}
residue LAT
{}
   group
   atom  C1   type CH1E  charge  0.0  end
   atom  D1   type HA    charge  0.0  end
   atom  C2   type CH1E  charge  0.0  end
   atom  D2   type HA    charge  0.0  end
   atom  C3   type CH1E  charge  0.0  end
   atom  D3   type HA    charge  0.0  end
   atom  C4   type CH1E  charge  0.0  end
   atom  D4   type HA    charge  0.0  end
   atom  C5   type CH1E  charge  0.0  end
   atom  D5   type HA    charge  0.0  end
   atom  C6   type CH2E  charge  0.0  end
   atom  D61  type HA    charge  0.0  end
   atom  D62  type HA    charge  0.0  end
   atom  O1   type OH1   charge  0.0  end
   atom  O2   type OH1   charge  0.0  end
   atom  DO2  type D     charge  0.0  end
   atom  O3   type OH1   charge  0.0  end
   atom  DO3  type D     charge  0.0  end
   atom  O4   type OH1   charge  0.0  end
   atom  DO4  type D     charge  0.0  end
   atom  O5   type OH1   charge  0.0  end
   atom  O6   type OH1   charge  0.0  end
   atom  DO6  type D     charge  0.0  end
   atom  C1'  type CH1E  charge  0.0  end
   atom  D1'  type HA    charge  0.0  end
   atom  C2'  type CH1E  charge  0.0  end
   atom  D2'  type HA    charge  0.0  end
   atom  C3'  type CH1E  charge  0.0  end
   atom  D3'  type HA    charge  0.0  end
   atom  C4'  type CH1E  charge  0.0  end
   atom  D4'  type HA    charge  0.0  end
   atom  C5'  type CH1E  charge  0.0  end
   atom  D5'  type HA    charge  0.0  end
   atom  C6'  type CH2E  charge  0.0  end
   atom  D6'1 type HA    charge  0.0  end
   atom  D6'2 type HA    charge  0.0  end
   atom  O2'  type OH1   charge  0.0  end
   atom  DO2' type D     charge  0.0  end
   atom  O3'  type OH1   charge  0.0  end
   atom  DO3' type D     charge  0.0  end
   atom  O5'  type OH1   charge  0.0  end
   atom  O6'  type OH1   charge  0.0  end
   atom  DO6' type D     charge  0.0  end
   atom  O1'  type OH1   charge  0.0  end
   atom  DO1' type D     charge  0.0  end
{}
   bond  C1   D1
   bond  C1   C2
   bond  C1   O1
   bond  C1   O5
   bond  C2   D2
   bond  C2   C3
   bond  C2   O2
   bond  C3   D3
   bond  C3   O3
   bond  C4   D4
   bond  C4   C5
```

```
   bond  C4   O4
   bond  C5   D5
   bond  C5   C6
   bond  C5   O5
   bond  C6   D61
   bond  C6   D62
   bond  C6   O6
   bond  O1   C4'
   bond  O6   DO6
   bond  C1'  D1'
   bond  C1'  C2'
   bond  C1'  O5'
   bond  C1'  O1'
   bond  C2'  D2'
   bond  C2'  C3'
   bond  C2'  O2'
   bond  C3'  D3'
   bond  C3'  C4'
   bond  C3'  O3'
   bond  C4'  D4'
   bond  C4'  C5'
   bond  C5'  D5'
   bond  C5'  C6'
   bond  C5'  O5'
   bond  C6'  D6'1
   bond  C6'  D6'2
   bond  C6'  O6'
   bond  O6'  DO6'
   bond  C3   C4
   bond  O2   DO2
   bond  O3   DO3
   bond  O4   DO4
   bond  O2'  DO2'
   bond  O3'  DO3'
   bond  O1'  DO1'
{}
   dihedral    D1   C1   C2   D2
   dihedral    D1   C1   C2   C3
   dihedral    D1   C1   C2   O2
   dihedral    D1   C1   O1   C4'
   dihedral    D1   C1   O5   C5
   dihedral    O1   C1   C2   D2
   dihedral    O1   C1   C2   C3
   dihedral    O1   C1   C2   O2
   dihedral    C2   C1   O1   C4'
   dihedral    O5   C1   C2   D2
   dihedral    O5   C1   C2   C3
   dihedral    O5   C1   C2   O2
   dihedral    C2   C1   O5   C5
   dihedral    C1   C2   C3   D3
   dihedral    C1   C2   C3   O3
   dihedral    C1   C2   C3   C4
   dihedral    C1   C2   O2   DO2
   dihedral    O5   C1   O1   C4'
   dihedral    O1   C1   O5   C5
   dihedral    C1   O1   C4'  C3'
   dihedral    C1   O1   C4'  D4'
   dihedral    C1   O1   C4'  C5'
   dihedral    C1   O5   C5   C4
   dihedral    C1   O5   C5   D5
   dihedral    C1   O5   C5   C6
   dihedral    D2   C2   C3   D3
   dihedral    D2   C2   C3   O3
   dihedral    D2   C2   C3   C4
   dihedral    D2   C2   O2   DO2
   dihedral    O2   C2   C3   D3
   dihedral    O2   C2   C3   O3
   dihedral    O2   C2   C3   C4
   dihedral    C3   C2   O2   DO2
   dihedral    C2   C3   O3   DO3
   dihedral    C2   C3   C4   D4
   dihedral    C2   C3   C4   C5
   dihedral    C2   C3   C4   O4
```

```
dihedral    D3   C3   O3   DO3
dihedral    D3   C3   C4   D4
dihedral    D3   C3   C4   C5
dihedral    D3   C3   C4   O4
dihedral    C4   C3   O3   DO3
dihedral    O3   C3   C4   D4
dihedral    O3   C3   C4   C5
dihedral    O3   C3   C4   O4
dihedral    D4   C4   C5   D5
dihedral    D4   C4   C5   C6
dihedral    D4   C4   C5   O5
dihedral    D4   C4   O4   DO4
dihedral    O4   C4   C5   D5
dihedral    O4   C4   C5   C6
dihedral    O4   C4   C5   O5
dihedral    C5   C4   O4   DO4
dihedral    D5   C5   C4   C3
dihedral    C6   C5   C4   C3
dihedral    C4   C5   C6   D61
dihedral    C4   C5   C6   D62
dihedral    C4   C5   C6   O6
dihedral    O5   C5   C4   C3
dihedral    C3   C4   O4   DO4
dihedral    D5   C5   C6   D61
dihedral    D5   C5   C6   D62
dihedral    D5   C5   C6   O6
dihedral    O5   C5   C6   D61
dihedral    O5   C5   C6   D62
dihedral    O5   C5   C6   O6
dihedral    C5   C6   O6   DO6
dihedral    D61  C6   O6   DO6
dihedral    D62  C6   O6   DO6
dihedral    O1   C4'  C3'  C2'
dihedral    O1   C4'  C3'  D3'
dihedral    O1   C4'  C3'  O3'
dihedral    O1   C4'  C5'  D5'
dihedral    O1   C4'  C5'  C6'
dihedral    O1   C4'  C5'  O5'
dihedral    D1'  C1'  C2'  D2'
dihedral    D1'  C1'  C2'  C3'
dihedral    D1'  C1'  C2'  O2'
dihedral    D1'  C1'  O5'  C5'
dihedral    D1'  C1'  O1'  DO1'
dihedral    O5'  C1'  C2'  D2'
dihedral    O5'  C1'  C2'  C3'
dihedral    O5'  C1'  C2'  O2'
dihedral    C2'  C1'  O5'  C5'
dihedral    O1'  C1'  C2'  D2'
dihedral    O1'  C1'  C2'  C3'
dihedral    O1'  C1'  C2'  O2'
dihedral    C2'  C1'  O1'  DO1'
dihedral    C1'  C2'  C3'  D3'
dihedral    C1'  C2'  C3'  C4'
dihedral    C1'  C2'  C3'  O3'
dihedral    C1'  C2'  O2'  DO2'
dihedral    O1'  C1'  O5'  C5'
dihedral    O5'  C1'  O1'  DO1'
dihedral    C1'  O5'  C5'  C4'
dihedral    C1'  O5'  C5'  D5'
dihedral    C1'  O5'  C5'  C6'
dihedral    D2'  C2'  C3'  D3'
dihedral    D2'  C2'  C3'  C4'
dihedral    D2'  C2'  C3'  O3'
dihedral    D2'  C2'  O2'  DO2'
dihedral    O2'  C2'  C3'  D3'
dihedral    O2'  C2'  C3'  C4'
dihedral    O2'  C2'  C3'  O3'
dihedral    C3'  C2'  O2'  DO2'
dihedral    C2'  C3'  C4'  D4'
dihedral    C2'  C3'  C4'  C5'
dihedral    C2'  C3'  O3'  DO3'
dihedral    D3'  C3'  C4'  D4'
dihedral    D3'  C3'  C4'  C5'
```

```
   dihedral    D3'  C3'  O3'  DO3'
   dihedral    O3'  C3'  C4'  D4'
   dihedral    O3'  C3'  C4'  C5'
   dihedral    C4'  C3'  O3'  DO3'
   dihedral    C3'  C4'  C5'  D5'
   dihedral    C3'  C4'  C5'  C6'
   dihedral    C3'  C4'  C5'  O5'
   dihedral    D4'  C4'  C5'  D5'
   dihedral    D4'  C4'  C5'  C6'
   dihedral    D4'  C4'  C5'  O5'
   dihedral    C4'  C5'  C6'  D6'1
   dihedral    C4'  C5'  C6'  D6'2
   dihedral    C4'  C5'  C6'  O6'
   dihedral    D5'  C5'  C6'  D6'1
   dihedral    D5'  C5'  C6'  D6'2
   dihedral    D5'  C5'  C6'  O6'
   dihedral    O5'  C5'  C6'  D6'1
   dihedral    O5'  C5'  C6'  D6'2
   dihedral    O5'  C5'  C6'  O6'
   dihedral    C5'  C6'  O6'  DO6'
   dihedral    D6'1 C6'  O6'  DO6'
   dihedral    D6'2 C6'  O6'  DO6'
{}
END {RESIdue LAT}
```

Table S3. The CNS parameter file employed for lactose.

```
remark Topology file for LAT, UR & FM 27/1-15
remark /home/senape/Refinement/Refinementforncns Thu Dec 4 14:56:47 CET 2014
set echo=false end
{}
{Force constants have been multiplied by a factor of    3.000}
bond CH1E HA     875.71      1.119
!bond CH1E CH1E   613.68      1.557
!bond CH1E OH1    1032.89      1.418
!bond CH1E OH1   1128.93      1.406
!bond CH1E CH2E   664.37      1.545
bond CH2E HA     952.38      1.106
!bond CH2E OH1   1183.25      1.401
!bond OH1  D     1778.69      0.949
{}
{Force constants have been multiplied by a factor of    3.000}
angle HA   CH1E CH1E  208.91     110.07
!angle HA   CH1E OH1    182.51     111.05
!angle CH1E CH1E OH1    340.30     110.17
angle CH1E CH1E CH1E  384.10     108.91
!angle CH1E CH1E OH1   316.89     109.90
!angle OH1   CH1E OH1    211.03      98.75
angle CH1E OH1   CH1E  484.52     114.73
angle HA   CH1E OH1   180.64     108.79
!angle CH1E OH1  D     209.69     107.11
!angle CH1E CH1E CH2E  420.52     112.54
angle HA   CH1E CH2E  159.55     109.39
angle CH2E CH1E OH1    400.29     105.81
angle CH1E CH2E HA    178.53     110.58
!angle CH1E CH2E OH1   376.19     113.22
angle HA   CH2E HA    114.54     108.05
angle HA   CH2E OH1   190.65     107.01
!angle CH2E OH1  D     203.60     107.38
angle OH1   CH1E OH1   287.06      98.44
{}
{Fconst = Orig_Fconst * 2 *    3.000 / ( n2 * n3 * Period^2 ) }
{           Atoms           Fconst Period Phase    Orig_Fconst n2 n3}
!dihe  HA   CH1E CH1E HA       2.70    3    0.00     !    36.48 3 3
!dihe  HA   CH1E CH1E CH1E     4.58    3    0.00     !    61.79 3 3
!dihe  HA   CH1E CH1E OH1      3.70    3    0.00     !    50.00 3 3
dihe  HA   CH1E OH1   CH1E     8.53    3    0.00     !    38.37 3 1
!dihe  OH1   CH1E CH1E HA       4.50    3    0.00     !    60.81 3 3
!dihe  OSH1  CH1E CH1E CH1E     8.73    3    0.00     !   117.84 3 3
```

```
!dihe  OH1   CH1E CH1E OH1       6.81    3    0.00     !    91.88 3 3
dihe  CH1E CH1E OH1   CH1E    16.33    3    0.00     !    73.47 3 1
!dihe  CH1E CH1E CH1E OH1      7.37    3    0.00     !    99.44 3 3
!dihe  CH1E CH1E CH1E CH1E     9.06    3    0.00     !   122.28 3 3
!dihe  CH1E CH1E OH1  D        1.01    3    0.00     !     4.54 3 1
dihe  OH1   CH1E OH1   CH1E    13.41    3    0.00     !    60.35 3 1
dihe  CH1E OH1   CH1E CH2E    17.13    3    0.00     !    77.08 1 3
!dihe  HA   CH1E OH1  D        0.67    3    0.00     !     3.00 3 1
!dihe  OH1  CH1E CH1E OH1      5.45    3    0.00     !    73.59 3 3
!dihe  HA   CH1E CH1E CH2E     4.52    3    0.00     !    61.07 3 3
!dihe  OH1  CH1E CH1E CH2E     7.01    3    0.00     !    94.66 3 3
!dihe  CH2E CH1E CH1E CH1E    10.32    3    0.00     !   139.30 3 3
!dihe  CH1E CH1E CH2E HA       4.74    3    0.00     !    63.95 3 3
!dihe  CH1E CH1E CH2E OH1      7.18    3    0.00     !    96.87 3 3
!dihe  HA   CH1E CH2E HA       2.63    3    0.00     !    35.49 3 3
!dihe  HA   CH1E CH2E OH1      3.59    3    0.00     !    48.42 3 3
dihe  OH1   CH1E CH2E HA       3.37    3    0.00     !    45.46 3 3
dihe  OH1   CH1E CH2E OH1      4.86    3    0.00     !    65.63 3 3
!dihe  CH1E CH2E OH1  D        0.92    3    0.00     !     4.15 3 1
!dihe  HA   CH2E OH1  D        0.63    3    0.00     !     2.85 3 1
dihe  OH1   CH1E CH1E CH2E     7.36    3    0.00     !    99.42 3 3
dihe  OH1   CH1E CH1E OH1       8.59    3    0.00     !   115.90 3 3
dihe  OH1  CH1E OH1   CH1E    14.25    3    0.00     !    64.12 3 1
dihe  OH1   CH1E OH1  D        1.23    3    0.00     !     5.55 3 1
{}
{* nonbonding parameter section *}
 !                  eps     sigma       eps(1:4) sigma(1:4)
 !                  (kcal/mol) (A)
 !                  ---------------------------------------
!nonbonded  CH1E    0.1200   3.7418      0.1000   3.3854 ! = C,    carbonyl carbon
nonbonded  HA      0.0000   2.0000      0.0000   2.0000 ! Dummy
!nonbonded  CH2E    0.1200   3.7418      0.1000   3.3854 ! = C,    carbonyl carbon
!nonbonded  OS      0.1591   2.8509      0.1591   2.8509 ! = O
!nonbonded  OH1     0.1591   2.8509      0.1591   2.8509 ! = O
!nonbonded  D       0.0000   2.0000      0.0000   2.0000 ! Dummy
```

Table S4. Coordinates (in PDB format) of the QM system after refinement of the galectin-3 preliminary structure with $w_X = 3$ and $w_N = 1$.

ATOM	1	HG	ARG	144	12.181	-11.230	4.475
ATOM	2	CD	ARG	144	12.552	-11.395	3.451
ATOM	3	DD1	ARG	144	11.744	-11.937	2.907
ATOM	4	DD2	ARG	144	13.420	-12.073	3.458
ATOM	5	NE	ARG	144	13.056	-10.137	2.865
ATOM	6	DE	ARG	144	14.099	-10.081	2.905
ATOM	7	CZ	ARG	144	12.382	-9.180	2.196
ATOM	8	NH1	ARG	144	11.021	-9.143	2.208
ATOM	9	DH11	ARG	144	10.494	-9.902	2.637
ATOM	10	DH12	ARG	144	10.513	-8.555	1.549
ATOM	11	NH2	ARG	144	13.069	-8.196	1.574
ATOM	12	DH21	ARG	144	14.119	-8.307	1.472
ATOM	13	DH22	ARG	144	12.577	-7.462	1.074
ATOM	14	HB	HIS	158	16.645	-1.597	1.069
ATOM	15	CG	HIS	158	16.414	-2.662	1.221
ATOM	16	ND1	HIS	158	15.233	-3.298	0.927
ATOM	17	DD1	HIS	158	14.364	-2.853	0.660
ATOM	18	CD2	HIS	158	17.316	-3.644	1.617
ATOM	19	DD2	HIS	158	18.353	-3.559	1.931
ATOM	20	CE1	HIS	158	15.458	-4.674	1.120
ATOM	21	DE1	HIS	158	14.681	-5.433	1.003
ATOM	22	NE2	HIS	158	16.690	-4.880	1.492
ATOM	23	HG	ARG	162	18.524	-9.978	6.361
ATOM	24	CD	ARG	162	19.390	-10.189	5.734
ATOM	25	DD1	ARG	162	19.444	-11.298	5.633
ATOM	26	DD2	ARG	162	20.322	-9.892	6.240
ATOM	27	NE	ARG	162	19.228	-9.377	4.508
ATOM	28	DE	ARG	162	18.277	-9.159	4.217
ATOM	29	CZ	ARG	162	20.188	-9.352	3.556
ATOM	30	NH1	ARG	162	21.334	-10.059	3.684
ATOM	31	DH11	ARG	162	21.620	-10.696	4.454
ATOM	32	DH12	ARG	162	22.071	-9.872	2.968
ATOM	33	NH2	ARG	162	20.026	-8.544	2.498
ATOM	34	DH21	ARG	162	19.154	-8.062	2.257
ATOM	35	DH22	ARG	162	20.718	-8.562	1.731
ATOM	36	HB	GLU	165	22.129	-13.518	7.508
ATOM	37	CG	GLU	165	22.836	-13.568	6.662
ATOM	38	DG1	GLU	165	22.306	-14.184	5.901
ATOM	39	DG2	GLU	165	23.730	-14.136	6.971
ATOM	40	CD	GLU	165	23.310	-12.185	6.323
ATOM	41	OE1	GLU	165	24.099	-11.686	7.157
ATOM	42	OE2	GLU	165	22.921	-11.629	5.240
ATOM	43	HA	ASN	174	21.308	-0.626	1.055
ATOM	44	CB	ASN	174	21.470	-1.610	0.571
ATOM	45	DB1	ASN	174	20.474	-1.908	0.174
ATOM	46	DB2	ASN	174	21.721	-2.356	1.350
ATOM	47	CG	ASN	174	22.640	-1.560	-0.396
ATOM	48	OD1	ASN	174	23.181	-0.543	-0.790
ATOM	49	ND2	ASN	174	23.124	-2.798	-0.761
ATOM	50	DD21	ASN	174	22.619	-3.661	-0.511
ATOM	51	DD22	ASN	174	23.914	-2.816	-1.398
ATOM	52	HB	TRP	181	21.821	-4.105	-4.137
ATOM	53	CG	TRP	181	20.807	-4.433	-4.074
ATOM	54	CD1	TRP	181	20.254	-5.557	-4.658
ATOM	55	DD1	TRP	181	20.738	-6.303	-5.289
ATOM	56	CD2	TRP	181	19.740	-3.744	-3.402
ATOM	57	NE1	TRP	181	18.894	-5.606	-4.389
ATOM	58	DE1	TRP	181	18.254	-6.305	-4.761
ATOM	59	CE2	TRP	181	18.548	-4.488	-3.599
ATOM	60	CE3	TRP	181	19.725	-2.582	-2.557
ATOM	61	DE3	TRP	181	20.626	-1.986	-2.399
ATOM	62	CZ2	TRP	181	17.315	-4.149	-3.004
ATOM	63	DZ2	TRP	181	16.415	-4.754	-3.154
ATOM	64	CZ3	TRP	181	18.499	-2.249	-1.962
ATOM	65	DZ3	TRP	181	18.459	-1.358	-1.319
ATOM	66	CH2	TRP	181	17.323	-3.024	-2.155
ATOM	67	DH2	TRP	181	16.395	-2.715	-1.666
ATOM	68	HB	GLU	184	25.178	-4.235	2.351
ATOM	69	CG	GLU	184	24.682	-4.778	1.544
ATOM	70	DG1	GLU	184	25.251	-4.518	0.623
ATOM	71	DG2	GLU	184	23.662	-4.390	1.370
ATOM	72	CD	GLU	184	24.507	-6.290	1.885
ATOM	73	OE1	GLU	184	25.155	-6.828	2.813
ATOM	74	OE2	GLU	184	23.606	-6.882	1.181
ATOM	75	HG	ARG	186	25.674	-5.830	6.870
ATOM	76	CD	ARG	186	26.294	-6.751	6.676
ATOM	77	DD1	ARG	186	26.336	-7.296	7.640
ATOM	78	DD2	ARG	186	27.337	-6.470	6.454

```
ATOM     79   NE  ARG   186       25.731  -7.426   5.510
ATOM     80   DE  ARG   186       25.758  -6.945   4.597
ATOM     81   CZ  ARG   186       25.122  -8.634   5.525
ATOM     82   NH1 ARG   186       25.022  -9.334   6.662
ATOM     83  DH11 ARG   186       25.457  -8.942   7.494
ATOM     84  DH12 ARG   186       24.590 -10.324   6.758
ATOM     85   NH2 ARG   186       24.635  -9.140   4.387
ATOM     86  DH21 ARG   186       24.704  -8.597   3.513
ATOM     87  DH22 ARG   186       24.122 -10.030   4.437
ATOM     88   C1  LAT   501       20.303  -8.758  -1.162
ATOM     89   D1  LAT   501       20.624  -8.452  -2.183
ATOM     90   C2  LAT   501       18.823  -9.123  -1.164
ATOM     91   D2  LAT   501       18.576  -9.569  -0.183
ATOM     92   C3  LAT   501       17.930  -7.893  -1.387
ATOM     93   D3  LAT   501       18.033  -7.590  -2.449
ATOM     94   C4  LAT   501       18.396  -6.726  -0.477
ATOM     95   D4  LAT   501       17.848  -5.806  -0.771
ATOM     96   C5  LAT   501       19.884  -6.461  -0.656
ATOM     97   D5  LAT   501       20.109  -6.194  -1.711
ATOM     98   C6  LAT   501       20.434  -5.341   0.266
ATOM     99   D61 LAT   501       19.939  -4.389   0.000
ATOM    100   D62 LAT   501       20.176  -5.596   1.316
ATOM    101   O1  LAT   501       21.035  -9.864  -0.735
ATOM    102   O2  LAT   501       18.587 -10.128  -2.154
ATOM    103   DO2 LAT   501       18.774  -9.734  -3.049
ATOM    104   O3  LAT   501       16.586  -8.247  -1.104
ATOM    105   DO3 LAT   501       15.949  -7.599  -1.505
ATOM    106   O4  LAT   501       18.059  -7.080   0.865
ATOM    107   DO4 LAT   501       17.517  -6.317   1.248
ATOM    108   O5  LAT   501       20.635  -7.648  -0.282
ATOM    109   O6  LAT   501       21.814  -5.172   0.096
ATOM    110   DO6 LAT   501       22.358  -5.915   0.522
ATOM    111   C1' LAT   501       24.835 -11.319  -0.904
ATOM    112   D1' LAT   501       24.282 -12.251  -0.649
ATOM    113   C2' LAT   501       24.662 -10.267   0.199
ATOM    114   D2' LAT   501       25.077  -9.307  -0.179
ATOM    115   C3' LAT   501       23.159 -10.137   0.454
ATOM    116   D3' LAT   501       22.779 -11.124   0.780
ATOM    117   C4' LAT   501       22.454  -9.729  -0.881
ATOM    118   D4' LAT   501       22.723  -8.686  -1.129
ATOM    119   C5' LAT   501       22.876 -10.709  -2.040
ATOM    120   D5' LAT   501       22.452 -11.710  -1.799
ATOM    121   C6' LAT   501       22.352 -10.270  -3.406
ATOM    122  D6'1 LAT   501       22.919 -10.836  -4.174
ATOM    123  D6'2 LAT   501       21.283 -10.543  -3.478
ATOM    124   O2' LAT   501       25.327 -10.645   1.406
ATOM    125  DO2' LAT   501       26.210 -10.987   1.141
ATOM    126   O3' LAT   501       22.910  -9.264   1.531
ATOM    127  DO3' LAT   501       23.284  -8.310   1.386
ATOM    128   O5' LAT   501       24.298 -10.772  -2.118
ATOM    129   O6' LAT   501       22.406  -8.861  -3.674
ATOM    130  DO6' LAT   501       23.144  -8.403  -3.199
ATOM    131   O1' LAT   501       26.197 -11.552  -1.032
ATOM    132  DO1' LAT   501       26.346 -12.371  -1.550
ATOM    133   O   DOD     1       15.636  -8.883   1.412
ATOM    134   D1  DOD     1       16.309  -8.725   2.104
ATOM    135   D2  DOD     1       16.091  -8.654   0.534
ATOM    136   O   DOD     2       15.725 -10.663   3.673
ATOM    137   D1  DOD     2       16.405 -11.079   3.061
ATOM    138   D2  DOD     2       15.755 -11.185   4.501
ATOM    139   O   DOD     3       14.476  -6.487  -2.046
ATOM    140   D1  DOD     3       14.781  -5.572  -1.867
ATOM    141   D2  DOD     3       14.342  -6.523  -3.018
ATOM    142   O   DOD     4       24.024 -12.686   2.861
ATOM    143   D1  DOD     4       24.490 -11.898   2.499
ATOM    144   D2  DOD     4       23.614 -12.389   3.710
ATOM    145   O   DOD     5       23.740  -6.536  -2.046
ATOM    146   D1  DOD     5       24.115  -6.687  -1.147
ATOM    147   D2  DOD     5       22.876  -6.114  -1.832
ATOM    148   O   DOD     6       19.263  -9.111  -4.667
ATOM    149   D1  DOD     6       19.253  -9.817  -5.348
ATOM    150   D2  DOD     6       20.218  -8.872  -4.558
ATOM    151   O   DOD     7       17.669 -11.560   2.004
ATOM    152   D1  DOD     7       18.484 -12.088   2.137
ATOM    153   D2  DOD     7       17.555 -11.469   1.032
END
```

Table S5. Coordinates of the QM system after refinement of the galectin-3 deposited structure (PDB entry 6EYM) with $w_X = 15$ and $w_N = 5$.

ATOM	1	HG	ARG	144	12.043	-11.320	4.497
ATOM	2	CD	ARG	144	12.291	-11.516	3.397
ATOM	3	DD1	ARG	144	11.360	-11.820	2.884
ATOM	4	DD2	ARG	144	13.051	-12.308	3.309
ATOM	5	NE	ARG	144	13.023	-10.201	2.898
ATOM	6	DE	ARG	144	14.060	-10.325	2.850
ATOM	7	CZ	ARG	144	12.417	-9.219	2.159
ATOM	8	NH1	ARG	144	11.026	-9.100	2.236
ATOM	9	DH11	ARG	144	10.514	-9.990	2.226
ATOM	10	DH12	ARG	144	10.585	-8.424	1.614
ATOM	11	NH2	ARG	144	13.141	-8.195	1.570
ATOM	12	DH21	ARG	144	14.188	-8.329	1.419
ATOM	13	DH22	ARG	144	12.639	-7.546	0.965
ATOM	14	HB	HIS	158	16.654	-1.617	1.068
ATOM	15	CG	HIS	158	16.335	-2.676	1.204
ATOM	16	ND1	HIS	158	15.181	-3.314	0.923
ATOM	17	DD1	HIS	158	14.303	-2.847	0.754
ATOM	18	CD2	HIS	158	17.349	-3.540	1.598
ATOM	19	DD2	HIS	158	18.403	-3.493	1.853
ATOM	20	CE1	HIS	158	15.511	-4.713	1.173
ATOM	21	DE1	HIS	158	14.715	-5.431	1.027
ATOM	22	NE2	HIS	158	16.710	-4.846	1.541
ATOM	23	HG	ARG	162	18.502	-10.037	6.364
ATOM	24	CD	ARG	162	19.407	-10.281	5.762
ATOM	25	DD1	ARG	162	19.604	-11.348	5.668
ATOM	26	DD2	ARG	162	20.278	-9.871	6.270
ATOM	27	NE	ARG	162	19.183	-9.356	4.504
ATOM	28	DE	ARG	162	18.358	-8.768	4.448
ATOM	29	CZ	ARG	162	20.157	-9.314	3.645
ATOM	30	NH1	ARG	162	21.405	-9.999	3.701
ATOM	31	DH11	ARG	162	21.568	-10.719	4.412
ATOM	32	DH12	ARG	162	22.123	-9.910	2.950
ATOM	33	NH2	ARG	162	20.022	-8.555	2.530
ATOM	34	DH21	ARG	162	19.184	-8.006	2.344
ATOM	35	DH22	ARG	162	20.765	-8.555	1.806
ATOM	36	HB	GLU	165	22.046	-13.480	7.485
ATOM	37	CG	GLU	165	22.716	-13.569	6.596
ATOM	38	DG1	GLU	165	22.115	-13.999	5.775
ATOM	39	DG2	GLU	165	23.506	-14.302	6.801
ATOM	40	CD	GLU	165	23.389	-12.293	6.418
ATOM	41	OE1	GLU	165	24.126	-11.794	7.308
ATOM	42	OE2	GLU	165	23.279	-11.799	5.266
ATOM	43	HA	ASN	174	21.308	-0.651	1.032
ATOM	44	CB	ASN	174	21.471	-1.661	0.577
ATOM	45	DB1	ASN	174	20.504	-1.960	0.129
ATOM	46	DB2	ASN	174	21.807	-2.288	1.405
ATOM	47	CG	ASN	174	22.660	-1.554	-0.406
ATOM	48	OD1	ASN	174	23.181	-0.541	-0.816
ATOM	49	ND2	ASN	174	23.151	-2.829	-0.794
ATOM	50	DD21	ASN	174	23.994	-2.823	-1.363
ATOM	51	DD22	ASN	174	22.524	-3.622	-0.586
ATOM	52	HB	TRP	181	21.799	-4.159	-4.152
ATOM	53	CG	TRP	181	20.789	-4.451	-4.064
ATOM	54	CD1	TRP	181	20.165	-5.547	-4.628
ATOM	55	DD1	TRP	181	20.689	-6.310	-5.220
ATOM	56	CD2	TRP	181	19.686	-3.735	-3.423
ATOM	57	NE1	TRP	181	18.806	-5.635	-4.402
ATOM	58	DE1	TRP	181	18.106	-6.294	-4.783
ATOM	59	CE2	TRP	181	18.496	-4.438	-3.529
ATOM	60	CE3	TRP	181	19.855	-2.589	-2.566
ATOM	61	DE3	TRP	181	20.754	-1.991	-2.457
ATOM	62	CZ2	TRP	181	17.254	-4.176	-2.990
ATOM	63	DZ2	TRP	181	16.371	-4.788	-3.083
ATOM	64	CZ3	TRP	181	18.563	-2.280	-1.999
ATOM	65	DZ3	TRP	181	18.455	-1.322	-1.457
ATOM	66	CH2	TRP	181	17.357	-3.016	-2.169
ATOM	67	DH2	TRP	181	16.449	-2.607	-1.740
ATOM	68	HB	GLU	184	25.221	-4.286	2.380
ATOM	69	CG	GLU	184	24.721	-4.755	1.569
ATOM	70	DG1	GLU	184	25.343	-4.424	0.711
ATOM	71	DG2	GLU	184	23.685	-4.418	1.308
ATOM	72	CD	GLU	184	24.511	-6.371	1.876
ATOM	73	OE1	GLU	184	25.193	-6.876	2.794
ATOM	74	OE2	GLU	184	23.603	-6.883	1.274
ATOM	75	HG	ARG	186	25.717	-5.859	6.921
ATOM	76	CD	ARG	186	26.327	-6.764	6.793
ATOM	77	DD1	ARG	186	26.235	-7.360	7.716
ATOM	78	DD2	ARG	186	27.390	-6.563	6.637
ATOM	79	NE	ARG	186	25.750	-7.405	5.563

```
ATOM     80  DE  ARG   186       25.655  -6.848   4.697
ATOM     81  CZ  ARG   186       25.132  -8.619   5.587
ATOM     82  NH1 ARG   186       25.094  -9.374   6.758
ATOM     83 DH11 ARG   186       25.432  -8.910   7.605
ATOM     84 DH12 ARG   186       24.630 -10.355   6.793
ATOM     85  NH2 ARG   186       24.669  -9.139   4.465
ATOM     86 DH21 ARG   186       24.712  -8.564   3.589
ATOM     87 DH22 ARG   186       24.111 -10.010   4.563
ATOM     88  C1  LAT   501       20.165  -8.804  -1.126
ATOM     89  D1  LAT   501       20.477  -8.470  -2.140
ATOM     90  C2  LAT   501       18.777  -9.166  -1.085
ATOM     91  D2  LAT   501       18.486  -9.705  -0.173
ATOM     92  C3  LAT   501       17.906  -7.999  -1.374
ATOM     93  D3  LAT   501       18.083  -7.717  -2.428
ATOM     94  C4  LAT   501       18.399  -6.855  -0.499
ATOM     95  D4  LAT   501       17.853  -5.943  -0.847
ATOM     96  C5  LAT   501       19.794  -6.538  -0.738
ATOM     97  D5  LAT   501       20.033  -6.282  -1.771
ATOM     98  C6  LAT   501       20.396  -5.359   0.249
ATOM     99  D61 LAT   501       19.917  -4.383   0.064
ATOM    100  D62 LAT   501       20.142  -5.686   1.279
ATOM    101  O1  LAT   501       21.048  -9.889  -0.715
ATOM    102  O2  LAT   501       18.709 -10.096  -2.237
ATOM    103  DO2 LAT   501       19.450  -9.838  -2.830
ATOM    104  O3  LAT   501       16.546  -8.293  -1.112
ATOM    105  DO3 LAT   501       15.945  -7.607  -1.500
ATOM    106  O4  LAT   501       18.094  -7.056   0.937
ATOM    107  DO4 LAT   501       17.523  -6.274   1.209
ATOM    108  O5  LAT   501       20.722  -7.617  -0.300
ATOM    109  O6  LAT   501       21.771  -5.192   0.114
ATOM    110  DO6 LAT   501       22.271  -6.022   0.347
ATOM    111  C1' LAT   501       24.830 -11.222  -0.950
ATOM    112  D1' LAT   501       24.455 -12.241  -0.739
ATOM    113  C2' LAT   501       24.626 -10.268   0.242
ATOM    114  D2' LAT   501       25.044  -9.279  -0.014
ATOM    115  C3' LAT   501       23.144 -10.174   0.357
ATOM    116  D3' LAT   501       22.714 -11.142   0.669
ATOM    117  C4' LAT   501       22.412  -9.669  -0.935
ATOM    118  D4' LAT   501       22.679  -8.657  -1.272
ATOM    119  C5' LAT   501       22.848 -10.862  -2.100
ATOM    120  D5' LAT   501       22.490 -11.877  -1.847
ATOM    121  C6' LAT   501       22.280 -10.248  -3.368
ATOM    122 D6'1 LAT   501       22.577 -10.853  -4.252
ATOM    123 D6'2 LAT   501       21.181 -10.208  -3.318
ATOM    124  O2' LAT   501       25.341 -10.750   1.426
ATOM    125 DO2' LAT   501       25.995 -10.071   1.690
ATOM    126  O3' LAT   501       22.922  -9.258   1.494
ATOM    127 DO3' LAT   501       23.249  -8.323   1.280
ATOM    128  O5' LAT   501       24.225 -10.677  -2.113
ATOM    129  O6' LAT   501       22.742  -8.877  -3.548
ATOM    130 DO6' LAT   501       23.715  -8.920  -3.400
ATOM    131  O1' LAT   501       26.197 -11.215  -1.241
ATOM    132 DO1' LAT   501       26.641 -11.760  -0.550
ATOM    133  O   DOD   614       23.932 -12.724   2.929
ATOM    134  D1  DOD   614       24.439 -11.989   2.517
ATOM    135  D2  DOD   614       23.762 -12.435   3.859
ATOM    136  O   DOD   648       15.625  -8.903   1.470
ATOM    137  D1  DOD   648       16.194  -8.838   2.267
ATOM    138  D2  DOD   648       16.217  -8.684   0.669
ATOM    139  O   DOD   655       16.727  -7.319  -5.293
ATOM    140  D1  DOD   655       16.599  -7.646  -6.203
ATOM    141  D2  DOD   655       16.080  -7.785  -4.726
ATOM    142  O   DOD   666       14.416  -6.409  -2.061
ATOM    143  D1  DOD   666       14.950  -5.680  -1.689
ATOM    144  D2  DOD   666       14.335  -6.223  -3.020
ATOM    145  O   DOD   668       15.730 -10.785   3.749
ATOM    146  D1  DOD   668       16.555 -11.189   3.394
ATOM    147  D2  DOD   668       15.526 -11.326   4.541
END
```

Table S6. Coordinates of the QM system after refinement of the galectin-3 deposited structure (PDB entry 6EYM) with $w_X = 7$ and $w_N = 7$.

ATOM	1	HG	ARG	144	12.073	-11.288	4.486
ATOM	2	CD	ARG	144	12.356	-11.468	3.403
ATOM	3	DD1	ARG	144	11.431	-11.826	2.908
ATOM	4	DD2	ARG	144	13.117	-12.265	3.336
ATOM	5	NE	ARG	144	13.018	-10.179	2.868
ATOM	6	DE	ARG	144	14.057	-10.283	2.815
ATOM	7	CZ	ARG	144	12.400	-9.196	2.162
ATOM	8	NH1	ARG	144	11.019	-9.136	2.201
ATOM	9	DH11	ARG	144	10.532	-10.041	2.166
ATOM	10	DH12	ARG	144	10.540	-8.429	1.651
ATOM	11	NH2	ARG	144	13.106	-8.179	1.558
ATOM	12	DH21	ARG	144	14.152	-8.309	1.387
ATOM	13	DH22	ARG	144	12.605	-7.535	0.947
ATOM	14	HB	HIS	158	16.650	-1.611	1.078
ATOM	15	CG	HIS	158	16.356	-2.665	1.228
ATOM	16	ND1	HIS	158	15.188	-3.307	0.927
ATOM	17	DD1	HIS	158	14.320	-2.830	0.748
ATOM	18	CD2	HIS	158	17.331	-3.587	1.608
ATOM	19	DD2	HIS	158	18.386	-3.531	1.849
ATOM	20	CE1	HIS	158	15.471	-4.696	1.158
ATOM	21	DE1	HIS	158	14.680	-5.411	1.008
ATOM	22	NE2	HIS	158	16.676	-4.850	1.545
ATOM	23	HG	ARG	162	18.506	-10.017	6.351
ATOM	24	CD	ARG	162	19.415	-10.257	5.769
ATOM	25	DD1	ARG	162	19.646	-11.315	5.712
ATOM	26	DD2	ARG	162	20.266	-9.836	6.288
ATOM	27	NE	ARG	162	19.190	-9.385	4.503
ATOM	28	DE	ARG	162	18.388	-8.764	4.460
ATOM	29	CZ	ARG	162	20.174	-9.319	3.620
ATOM	30	NH1	ARG	162	21.382	-10.040	3.694
ATOM	31	DH11	ARG	162	21.537	-10.710	4.452
ATOM	32	DH12	ARG	162	22.149	-9.940	2.999
ATOM	33	NH2	ARG	162	20.019	-8.542	2.532
ATOM	34	DH21	ARG	162	19.163	-8.019	2.366
ATOM	35	DH22	ARG	162	20.744	-8.550	1.786
ATOM	36	HB	GLU	165	22.079	-13.492	7.474
ATOM	37	CG	GLU	165	22.785	-13.578	6.617
ATOM	38	DG1	GLU	165	22.248	-14.094	5.806
ATOM	39	DG2	GLU	165	23.616	-14.234	6.885
ATOM	40	CD	GLU	165	23.398	-12.260	6.401
ATOM	41	OE1	GLU	165	24.100	-11.774	7.327
ATOM	42	OE2	GLU	165	23.232	-11.736	5.270
ATOM	43	HA	ASN	174	21.307	-0.642	1.039
ATOM	44	CB	ASN	174	21.467	-1.642	0.571
ATOM	45	DB1	ASN	174	20.510	-1.950	0.112
ATOM	46	DB2	ASN	174	21.817	-2.255	1.402
ATOM	47	CG	ASN	174	22.656	-1.561	-0.419
ATOM	48	OD1	ASN	174	23.182	-0.535	-0.813
ATOM	49	ND2	ASN	174	23.130	-2.821	-0.791
ATOM	50	DD21	ASN	174	23.991	-2.818	-1.332
ATOM	51	DD22	ASN	174	22.491	-3.604	-0.583
ATOM	52	HB	TRP	181	21.808	-4.155	-4.141
ATOM	53	CG	TRP	181	20.780	-4.464	-4.054
ATOM	54	CD1	TRP	181	20.177	-5.555	-4.641
ATOM	55	DD1	TRP	181	20.714	-6.310	-5.228
ATOM	56	CD2	TRP	181	19.694	-3.732	-3.418
ATOM	57	NE1	TRP	181	18.808	-5.620	-4.410
ATOM	58	DE1	TRP	181	18.107	-6.290	-4.776
ATOM	59	CE2	TRP	181	18.498	-4.465	-3.564
ATOM	60	CE3	TRP	181	19.823	-2.591	-2.570
ATOM	61	DE3	TRP	181	20.735	-2.016	-2.451
ATOM	62	CZ2	TRP	181	17.269	-4.167	-2.980
ATOM	63	DZ2	TRP	181	16.373	-4.759	-3.055
ATOM	64	CZ3	TRP	181	18.552	-2.239	-1.994
ATOM	65	DZ3	TRP	181	18.455	-1.278	-1.461
ATOM	66	CH2	TRP	181	17.365	-2.993	-2.180
ATOM	67	DH2	TRP	181	16.441	-2.608	-1.776
ATOM	68	HB	GLU	184	25.221	-4.283	2.379
ATOM	69	CG	GLU	184	24.706	-4.776	1.567
ATOM	70	DG1	GLU	184	25.357	-4.438	0.736
ATOM	71	DG2	GLU	184	23.676	-4.433	1.269
ATOM	72	CD	GLU	184	24.519	-6.337	1.897
ATOM	73	OE1	GLU	184	25.220	-6.861	2.802
ATOM	74	OE2	GLU	184	23.618	-6.884	1.277
ATOM	75	HG	ARG	186	25.687	-5.851	6.913
ATOM	76	CD	ARG	186	26.288	-6.770	6.786
ATOM	77	DD1	ARG	186	26.174	-7.378	7.698
ATOM	78	DD2	ARG	186	27.353	-6.575	6.673
ATOM	79	NE	ARG	186	25.747	-7.399	5.543
ATOM	80	DE	ARG	186	25.637	-6.831	4.683

```
ATOM     81  CZ  ARG   186       25.152  -8.613   5.568
ATOM     82  NH1 ARG   186       25.102  -9.360   6.722
ATOM     83 DH11 ARG   186       25.448  -8.909   7.578
ATOM     84 DH12 ARG   186       24.601 -10.325   6.760
ATOM     85  NH2 ARG   186       24.641  -9.122   4.460
ATOM     86 DH21 ARG   186       24.715  -8.568   3.571
ATOM     87 DH22 ARG   186       24.099 -10.000   4.578
ATOM     88  C1  LAT   501       20.216  -8.784  -1.135
ATOM     89  D1  LAT   501       20.558  -8.455  -2.142
ATOM     90  C2  LAT   501       18.812  -9.162  -1.116
ATOM     91  D2  LAT   501       18.536  -9.673  -0.180
ATOM     92  C3  LAT   501       17.916  -7.974  -1.365
ATOM     93  D3  LAT   501       18.083  -7.697  -2.422
ATOM     94  C4  LAT   501       18.399  -6.830  -0.503
ATOM     95  D4  LAT   501       17.874  -5.911  -0.861
ATOM     96  C5  LAT   501       19.818  -6.530  -0.690
ATOM     97  D5  LAT   501       20.078  -6.270  -1.714
ATOM     98  C6  LAT   501       20.365  -5.368   0.250
ATOM     99  D61 LAT   501       19.877  -4.396   0.076
ATOM    100  D62 LAT   501       20.140  -5.679   1.292
ATOM    101  O1  LAT   501       21.038  -9.893  -0.728
ATOM    102  O2  LAT   501       18.689 -10.102  -2.230
ATOM    103  DO2 LAT   501       19.452  -9.910  -2.822
ATOM    104  O3  LAT   501       16.545  -8.308  -1.091
ATOM    105  DO3 LAT   501       15.933  -7.641  -1.487
ATOM    106  O4  LAT   501       18.085  -7.052   0.926
ATOM    107  DO4 LAT   501       17.505  -6.281   1.204
ATOM    108  O5  LAT   501       20.711  -7.633  -0.294
ATOM    109  O6  LAT   501       21.748  -5.195   0.097
ATOM    110  DO6 LAT   501       22.238  -6.030   0.324
ATOM    111  C1' LAT   501       24.853 -11.204  -0.958
ATOM    112  D1' LAT   501       24.494 -12.224  -0.724
ATOM    113  C2' LAT   501       24.619 -10.262   0.219
ATOM    114  D2' LAT   501       25.033  -9.260  -0.001
ATOM    115  C3' LAT   501       23.139 -10.154   0.393
ATOM    116  D3' LAT   501       22.733 -11.132   0.715
ATOM    117  C4' LAT   501       22.421  -9.696  -0.902
ATOM    118  D4' LAT   501       22.690  -8.673  -1.228
ATOM    119  C5' LAT   501       22.828 -10.820  -2.051
ATOM    120  D5' LAT   501       22.465 -11.834  -1.794
ATOM    121  C6' LAT   501       22.300 -10.280  -3.368
ATOM    122 D6'1 LAT   501       22.673 -10.913  -4.199
ATOM    123 D6'2 LAT   501       21.198 -10.264  -3.386
ATOM    124  O2' LAT   501       25.308 -10.752   1.404
ATOM    125 DO2' LAT   501       26.018 -10.125   1.656
ATOM    126  O3' LAT   501       22.911  -9.250   1.484
ATOM    127 DO3' LAT   501       23.252  -8.321   1.271
ATOM    128  O5' LAT   501       24.226 -10.696  -2.120
ATOM    129  O6' LAT   501       22.721  -8.909  -3.556
ATOM    130 DO6' LAT   501       23.693  -8.920  -3.389
ATOM    131  O1' LAT   501       26.228 -11.232  -1.250
ATOM    132 DO1' LAT   501       26.643 -11.846  -0.598
ATOM    133  O   DOD   614       23.902 -12.712   2.881
ATOM    134  D1  DOD   614       24.409 -11.987   2.449
ATOM    135  D2  DOD   614       23.780 -12.412   3.813
ATOM    136  O   DOD   648       15.649  -8.902   1.461
ATOM    137  D1  DOD   648       16.173  -8.913   2.290
ATOM    138  D2  DOD   648       16.282  -8.678   0.692
ATOM    139  O   DOD   655       16.684  -7.273  -5.276
ATOM    140  D1  DOD   655       16.611  -7.664  -6.166
ATOM    141  D2  DOD   655       16.048  -7.740  -4.697
ATOM    142  O   DOD   666       14.423  -6.447  -2.029
ATOM    143  D1  DOD   666       14.993  -5.773  -1.615
ATOM    144  D2  DOD   666       14.356  -6.211  -2.978
ATOM    145  O   DOD   668       15.739 -10.769   3.722
ATOM    146  D1  DOD   668       16.608 -11.174   3.486
ATOM    147  D2  DOD   668       15.410 -11.334   4.452
END
```

Table S7. Coordinates of the QM system after refinement of subunit A of LPMO with $w_X = 1$ and $w_N = 1$.

```
ATOM      1  CA  HIS A  32      -8.346 -16.854  15.319
ATOM      2  DA  HIS A  32      -7.300 -16.806  15.708
ATOM      3  CB  HIS A  32      -8.875 -18.064  14.537
ATOM      4  DB1 HIS A  32      -8.482 -18.035  13.506
ATOM      5  DB2 HIS A  32      -9.977 -17.939  14.484
ATOM      6  CG  HIS A  32      -8.543 -19.375  15.171
ATOM      7  ND1 HIS A  32      -8.848 -19.585  16.507
ATOM      8  CD2 HIS A  32      -7.955 -20.521  14.664
ATOM      9  DD2 HIS A  32      -7.554 -20.773  13.681
ATOM     10  CE1 HIS A  32      -8.425 -20.805  16.835
ATOM     11  DE1 HIS A  32      -8.565 -21.264  17.816
ATOM     12  NE2 HIS A  32      -7.882 -21.398  15.734
ATOM     13  DE2 HIS A  32      -7.478 -22.331  15.711
ATOM     14  HJ  HIS A  32      -8.386 -15.975  14.669
ATOM     15  N   HIS A  32      -9.198 -16.581  16.477
ATOM     16  DT1 HIS A  32     -10.154 -16.350  16.174
ATOM     17  DT2 HIS A  32      -8.851 -15.751  16.978
ATOM     18  HA  HIS A 109     -12.283 -13.801  20.932
ATOM     19  CB  HIS A 109     -11.392 -14.427  21.223
ATOM     20  DB1 HIS A 109     -11.496 -14.517  22.332
ATOM     21  DB2 HIS A 109     -10.471 -13.837  21.071
ATOM     22  CG  HIS A 109     -11.270 -15.664  20.393
ATOM     23  ND1 HIS A 109     -12.250 -16.639  20.340
ATOM     24  DD1 HIS A 109     -13.086 -16.662  20.920
ATOM     25  CD2 HIS A 109     -10.317 -16.079  19.483
ATOM     26  DD2 HIS A 109      -9.365 -15.604  19.241
ATOM     27  CE1 HIS A 109     -11.896 -17.576  19.405
ATOM     28  DE1 HIS A 109     -12.470 -18.471  19.166
ATOM     29  NE2 HIS A 109     -10.733 -17.240  18.858
ATOM     30  HA  PHE A 164      -2.066 -15.128  18.506
ATOM     31  CB  PHE A 164      -3.290 -14.829  17.624
ATOM     32  DB1 PHE A 164      -2.991 -14.863  16.576
ATOM     33  DB2 PHE A 164      -3.680 -13.843  17.869
ATOM     34  CG  PHE A 164      -4.380 -15.819  17.825
ATOM     35  CD1 PHE A 164      -5.223 -15.740  18.915
ATOM     36  DD1 PHE A 164      -5.111 -14.929  19.620
ATOM     37  DD2 PHE A 164      -4.526 -16.885  16.954
ATOM     38  DD2 PHE A 164      -3.865 -16.977  16.105
ATOM     39  CE1 PHE A 164      -6.183 -16.706  19.134
ATOM     40  DE1 PHE A 164      -6.839 -16.629  19.988
ATOM     41  CE2 PHE A 164      -5.485 -17.838  17.175
ATOM     42  DE2 PHE A 164      -5.592 -18.663  16.486
ATOM     43  CZ  PHE A 164      -6.304 -17.757  18.277
ATOM     44  DZ  PHE A 164      -7.081 -18.492  18.429
ATOM     45 CU+2 CU2 A 201      -9.674 -18.346  17.682
ATOM     46  O1  PER A 202      -8.982 -19.499  19.368
ATOM     47  O2  PER A 202     -10.029 -20.333  19.482
ATOM     48  O   DOD A 301     -11.528 -19.945  17.333
ATOM     49  D1  DOD A 301     -11.141 -19.095  17.159
ATOM     50  D2  DOD A 301     -12.467 -19.813  17.393
END
```

Table S7. Coordinates of the QM system after refinement of subunit B of LPMO with $w_X = 1$ and $w_N = 1$ and the N-terminus modelled as –ND_2.

```
ATOM      1  CA  HIS B  32     -10.707 -17.222  34.542
ATOM      2  DA  HIS B  32      -9.746 -17.476  35.049
ATOM      3  CB  HIS B  32     -10.710 -16.362  33.273
ATOM      4  DB1 HIS B  32     -10.622 -15.294  33.540
ATOM      5  DB2 HIS B  32     -11.686 -16.513  32.768
ATOM      6  CG  HIS B  32      -9.569 -16.767  32.372
ATOM      7  ND1 HIS B  32      -9.429 -18.117  32.060
ATOM      8  CD2 HIS B  32      -8.488 -16.088  31.792
ATOM      9  DD2 HIS B  32      -8.189 -15.035  31.785
ATOM     10  CE1 HIS B  32      -8.304 -18.273  31.362
ATOM     11  DE1 HIS B  32      -7.982 -19.256  31.001
ATOM     12  NE2 HIS B  32      -7.689 -17.057  31.214
ATOM     13  DE2 HIS B  32      -6.781 -16.897  30.783
ATOM     14  HJ  HIS B  32     -11.234 -16.684  35.315
ATOM     15  N   HIS B  32     -11.347 -18.514  34.311
ATOM     16  DT1 HIS B  32     -12.333 -18.400  34.027
ATOM     17  DT2 HIS B  32     -11.357 -19.060  35.185
ATOM     18  HA  HIS B 109     -15.023 -23.390  34.841
ATOM     19  CB  HIS B 109     -13.937 -23.614  34.761
ATOM     20  DB1 HIS B 109     -13.910 -24.711  34.545
ATOM     21  DB2 HIS B 109     -13.474 -23.508  35.761
ATOM     22  CG  HIS B 109     -13.215 -22.662  33.873
ATOM     23  ND1 HIS B 109     -13.526 -22.497  32.526
ATOM     24  DD1 HIS B 109     -14.227 -23.047  32.034
ATOM     25  CD2 HIS B 109     -12.204 -21.733  34.114
ATOM     26  DD2 HIS B 109     -11.673 -21.541  35.047
ATOM     27  CE1 HIS B 109     -12.714 -21.544  31.996
ATOM     28  DE1 HIS B 109     -12.703 -21.245  30.948
ATOM     29  NE2 HIS B 109     -11.930 -21.048  32.950
ATOM     30  HA  PHE B 164      -5.821 -20.212  38.964
ATOM     31  CB  PHE B 164      -7.119 -19.453  38.686
ATOM     32  DB1 PHE B 164      -6.954 -18.388  38.850
ATOM     33  DB2 PHE B 164      -7.902 -19.818  39.348
ATOM     34  CG  PHE B 164      -7.573 -19.641  37.289
ATOM     35  CD1 PHE B 164      -8.213 -20.804  36.915
ATOM     36  DD1 PHE B 164      -8.449 -21.550  37.659
ATOM     37  CD2 PHE B 164      -7.241 -18.723  36.316
ATOM     38  DD2 PHE B 164      -6.711 -17.822  36.587
ATOM     39  CE1 PHE B 164      -8.549 -21.022  35.590
ATOM     40  DE1 PHE B 164      -9.073 -21.923  35.307
ATOM     41  CE2 PHE B 164      -7.584 -18.948  34.997
ATOM     42  DE2 PHE B 164      -7.342 -18.212  34.245
ATOM     43  CZ  PHE B 164      -8.226 -20.092  34.643
ATOM     44  DZ  PHE B 164      -8.516 -20.249  33.615
ATOM     45 CU+2 CU2 B 201     -10.613 -19.618  32.584
ATOM     46  O1  PER B 202      -9.339 -21.946  31.875
ATOM     47  O2  PER B 202      -9.682 -20.773  31.226
ATOM     48  O   DOD B 307     -11.960 -18.877  30.792
ATOM     49  D1  DOD B 307     -11.129 -18.693  30.369
ATOM     50  D2  DOD B 307     -12.576 -19.100  30.103
END
```

Table S7. Coordinates of the QM system after refinement of subunit B of LPMO with w_X = 1 and w_N = 1 and the N-terminus modelled as –ND$^-$.

```
ATOM      1  CA  HIS B  32     -10.721 -17.273  34.529
ATOM      2  DA  HIS B  32      -9.741 -17.474  35.039
ATOM      3  CB  HIS B  32     -10.699 -16.357  33.257
ATOM      4  DB1 HIS B  32     -10.575 -15.276  33.503
ATOM      5  DB2 HIS B  32     -11.681 -16.500  32.763
ATOM      6  CG  HIS B  32      -9.568 -16.787  32.354
ATOM      7  ND1 HIS B  32      -9.412 -18.130  32.031
ATOM      8  CD2 HIS B  32      -8.477 -16.112  31.794
ATOM      9  DD2 HIS B  32      -8.179 -15.058  31.804
ATOM     10  CE1 HIS B  32      -8.286 -18.287  31.347
ATOM     11  DE1 HIS B  32      -7.983 -19.268  30.967
ATOM     12  NE2 HIS B  32      -7.664 -17.075  31.222
ATOM     13  DE2 HIS B  32      -6.735 -16.912  30.835
ATOM     14  HJ  HIS B  32     -11.238 -16.705  35.313
ATOM     15  N   HIS B  32     -11.342 -18.566  34.327
ATOM     16  DT1 HIS B  32     -12.334 -18.388  34.068
ATOM     17  HA  HIS B 109     -15.017 -23.382  34.838
ATOM     18  CB  HIS B 109     -13.923 -23.593  34.749
ATOM     19  DB1 HIS B 109     -13.892 -24.701  34.565
ATOM     20  DB2 HIS B 109     -13.452 -23.455  35.744
ATOM     21  CG  HIS B 109     -13.234 -22.641  33.843
ATOM     22  ND1 HIS B 109     -13.592 -22.507  32.497
ATOM     23  DD1 HIS B 109     -14.494 -22.838  32.135
ATOM     24  CD2 HIS B 109     -12.200 -21.725  34.037
ATOM     25  DD2 HIS B 109     -11.660 -21.457  34.945
ATOM     26  CE1 HIS B 109     -12.801 -21.538  31.939
ATOM     27  DE1 HIS B 109     -12.805 -21.260  30.883
ATOM     28  NE2 HIS B 109     -11.959 -21.075  32.856
ATOM     29  HA  PHE B 164      -5.821 -20.212  38.964
ATOM     30  CB  PHE B 164      -7.119 -19.453  38.686
ATOM     31  DB1 PHE B 164      -6.954 -18.388  38.850
ATOM     32  DB2 PHE B 164      -7.902 -19.818  39.348
ATOM     33  CG  PHE B 164      -7.573 -19.641  37.289
ATOM     34  CD1 PHE B 164      -8.213 -20.804  36.915
ATOM     35  DD1 PHE B 164      -8.449 -21.550  37.659
ATOM     36  CD2 PHE B 164      -7.241 -18.723  36.316
ATOM     37  DD2 PHE B 164      -6.711 -17.822  36.587
ATOM     38  CE1 PHE B 164      -8.549 -21.022  35.590
ATOM     39  DE1 PHE B 164      -9.073 -21.923  35.307
ATOM     40  CE2 PHE B 164      -7.584 -18.948  34.997
ATOM     41  DE2 PHE B 164      -7.342 -18.212  34.245
ATOM     42  CZ  PHE B 164      -8.226 -20.092  34.643
ATOM     43  DZ  PHE B 164      -8.516 -20.249  33.615
ATOM     44 CU+2 CU2 B 201     -10.617 -19.605  32.590
ATOM     45  O1  PER B 202      -9.319 -21.916  31.906
ATOM     46  O2  PER B 202      -9.682 -20.749  31.238
ATOM     47  O   DOD B 307     -11.960 -18.877  30.792
ATOM     48  D1  DOD B 307     -11.129 -18.693  30.369
ATOM     49  D2  DOD B 307     -12.576 -19.100  30.103
END
```
